# Reversible acetylation of ribosomal protein S1 serves as a smart switch for *Salmonella* to rapidly adapt to host stress

**DOI:** 10.1093/nar/gkaf252

**Published:** 2025-04-01

**Authors:** Yi-Lin Shen, Tian-Xian Liu, Lei Xu, Bang-Ce Ye, Ying Zhou

**Affiliations:** Laboratory of Biosystems and Microanalysis, State Key Laboratory of Bioreactor Engineering, East China University of Science and Technology, Shanghai 200237, China; Laboratory of Biosystems and Microanalysis, State Key Laboratory of Bioreactor Engineering, East China University of Science and Technology, Shanghai 200237, China; Laboratory of Biosystems and Microanalysis, State Key Laboratory of Bioreactor Engineering, East China University of Science and Technology, Shanghai 200237, China; Laboratory of Biosystems and Microanalysis, State Key Laboratory of Bioreactor Engineering, East China University of Science and Technology, Shanghai 200237, China; Laboratory of Biosystems and Microanalysis, State Key Laboratory of Bioreactor Engineering, East China University of Science and Technology, Shanghai 200237, China

## Abstract

Reprogramming metabolic pathways is crucial for pathogens survival in the lethal environments. Here, we present a mechanism by which *Salmonella* can rapidly respond to the external environment at the translational level; namely, the dynamic acetylation changes at the K247 site of ribosomal protein S1 could modulate the different mRNAs translation to adapt to distinct infection stages. We uncovered that S1^K247^ preferentially recruits mRNAs associated with flagellum assembly, sulfur metabolism, and SPI-1 T3SS. Conversely, S1^K247Ac^ catalyzed by Pat favors the mRNAs linked to arginine biosynthesis, contributing to the activation of ArgR regulating SPI-2 virulence factors and enabling survival and replication within macrophages. Notably, a K247 acetyl-mimetic mutant strain exhibited increased virulence both *ex vivo* and *in vivo*. This mechanism not only aids in further understanding how the pathogen survives in complex environment but also facilitates in identifying new targets and pathways to eliminating pathogenic bacteria.

## Graphical Abstract

**Graphical Abstract:**
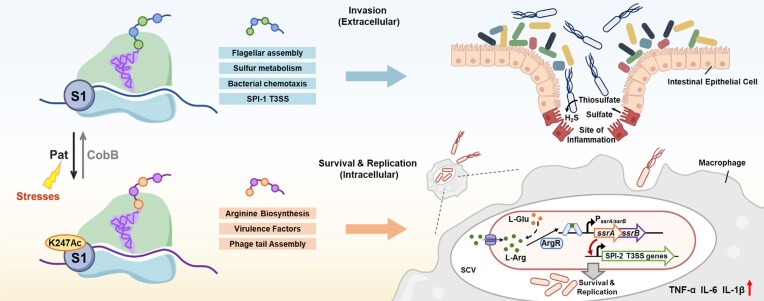

## Introduction

The success of intracellular pathogens is critically dependent on their capacity to rapidly sense and overcome challenges posed by the host environment. *Salmonella*, a typical representative of foodborne pathogens, demonstrates prowess in navigating adverse conditions. Upon invasion, *Salmonella* triggers host cells to unleash an array of defensive substances, such as reactive oxygen/nitrogen species [1], antibacterial peptides [2], lysosomes [3], low pH [4], and Mg^2+^ depletion [5], and *Salmonella* deals with them by activating the distinct gene program tailored to each stress. Bacteria commonly utilize two-component systems (TCSs) as regulatory circuits to sense and respond to environmental changes and stress conditions. A typical TCS consists of sensor histidine kinase (HK) and response regulator (RR). By transferring phosphate groups between these components, TCS enable signal sensing and regulation of downstream target genes. For instance, transcriptional regulator CadC and AdiY regulate *cadBA* and *adiAC* expression respectively, mitigating low-acid stress through proton depletion [6–8]. Within macrophages, *Salmonella* combats excess Fe^3+^ and acidic pH by activating PmrA–PmrB to induce *arnBCADTEF* transcription, producing L-4-aminoarabinose (L-Ara4N) for evading antimicrobial agents and host immunity [9, 10]. The PhoP–PhoQ system coordinates to diverse challenges, overseeing *mgtABC* transcription to combat Mg^2+^ depletion and maintain ribosomal assembly efficiency against reactive nitrogen species [11, 12]. Acidic pH triggers PhoP activation, upregulating *pagCDOK* transcription for virulence protein production [13]. The SsrA–SsrB system senses the acidic environment within the host cells and regulates the expression of *Salmonella* pathogenicity island 2 (SPI-2) to facilitate intracellular survival. SsrA, the sensor kinase, detects vacuolar acidic pH, while SsrB, the RR, activates SPI-2 genes. This pH-sensitive switch enables the pathogen to adapt to the intracellular environment and promote virulence [14]. Additional TCSs, including ArcAB [15], OmpR/EnvZ [16], and NarLX [17], respond to oxygen, osmolarity, and nitrate/nitrite, respectively, further highlighting the key role of transcriptional factors in rapidly adapting to environmental stresses.

In contemporary studies, a growing body of evidence suggests that living organisms not only rely on transcriptional regulation to adapt to external environmental fluctuations but also exhibit swift responses to stress through modulation of translation preferences [18]. Ribosome heterogeneity enables preferential translation of distinct subsets of messenger RNAs (mRNAs) tailored to specific stressors [19]. The heterogeneity encompasses variations in ribosomal protein composition [19], diversity in ribosomal RNA [20], activity of ribosome-associated factors [21], and chemical modifications of ribosomal proteins and RNAs [22, 23]. Recent researches have revealed that since initiation represents the bottleneck of the translation process [24], stress-induced modifications on eukaryotic initiation factors (eIFs) can dynamically reprogram protein synthesis by manipulating ribosomal activities in eukaryotes. For instance, Xin *et al.* demonstrated that de-O-GlcNAcylation induced by amino acid deprivation led to eIF3 retention on elongating 80S ribosomes, transforming eIF3 into a molecular switch that sustains protein synthesis activity [25]. Meanwhile, Lamper *et al.* revealed that phosphorylation-mediated activation of eIF3d near the cap-binding site rapidly redirected cellular protein synthesis towards factors crucial for glucose homeostasis [26]. However, the mechanism by which ribosome heterogeneity, resulting from post-translational modifications (PTM), directly regulates protein synthesis in bacteria remains poorly understood. Acetylation, as a key PTM, plays a crucial role in the precise regulation of various physiological processes in prokaryotic organisms [27]. The dynamic balance between enzymatic or nonenzymatic acetylation and deacetylation is tightly regulated by acetyltransferases, such as Pat, or acetyl donors like Ac-CoA, or AcP, and the NAD-dependent protein deacetylase CobB [28]. Our investigations have revealed the importance of acetylated S1 in ribosome-mediated gene expression regulation to confront environmental stresses in *Escherichia coli* MG1655 [29, 30]. S1, the largest ribosomal protein in the bacterial 30S subunit, is indispensable for translation initiation in bacteria. By binding to the upstream region of the Shine–Dalgarno sequence, S1 disrupts mRNA secondary structures, facilitating entry into the ribosome’s decoding channel [31–33]. Consequently, modulating S1’s affinity for ribosomes and mRNAs emerges as a universal strategy for bacteria to swiftly respond to external cues.

In this study, we proposed a mechanism by which the dynamic acetylation state at K247 in protein S1 orchestrates the virulence of *Salmonella typhimurium*. This regulatory process involves precise modulation of protein synthesis, driven by ribosomal heterogeneity induced by acetylation at K247 in S1. Through this selective recruitment of specific subsets of mRNAs for translation, *S. typhimurium* quickly fine-tunes its virulence responses at different infection stages. Our findings suggest that the ability of *Salmonella* to modulate the acetylation status of S1 at position 247 represents an adaptive and sophisticated strategy to navigate the challenges posed by its ever-changing external environment.

## Materials and methods

### Bacterial strains, plasmids, and primers

*Salmonella typhimurium* 14028S wild-type (WT) and genetically modified strains were used in this study. Deletions were constructed utilizing homologous recombination based on λ-Red system [34]. Given that *rpsA* is an essential gene of bacteria, we initially introduced a plasmid, under the control of the Pj23110 promoter, into wild *S. typhimurium* 14028S to constitutively overexpress protein S1 prior to homologous recombination. Subsequently, we employed the apramycin resistance gene (*Apr^R^*) to facilitate the deletion of the *rpsA* gene from the bacterial genome. Antibiotic resistance gene was generated by using primers with 50-nt homology extensions. Site-specific mutation of expressed protein was performed by *Fast* Mutagenesis System (FM111-01, TransGen Biotech, China). All strains, plasmids, and primers used in this study were described in Supplementary Tables S1 and S2.

### Growth conditions

Luria–Bertani broth (LB) was used as a rich medium, and M9 and 3-(N-Morpholino)propanesulfonic acid （MOPS）-salts medium was used as nutrition-restricted medium. Agar plates contained 1.5% (w/v) agar and cultural medium was supplemented with antibiotics as required. The working concentrations antibiotics were 100 μg/ml of ampicillin, 25 μg/ml of chloramphenicol, 50 μg/ml of kanamycin, 50 μg /ml of apramycin, and 100 μg /ml of streptomycin, respectively. Growth was monitored by measuring the optical density at 600 nm (OD_600_). All the chemical reagents were purchased from Macklin reagent (China). LB medium and agar were obtained from Generay Biotech (China). All the antibiotics were purchased from Solarbio (China).

### Overexpression and purification of proteins

For purification of Pat, CobB, ArgR, S1, and its derivative mutants, expression vectors were constructed and verified by sequencing. All the constructed plasmids were introduced into *E. coli* strain BL21 for *in vitro* experiment, and into *S. typhimurium* 14028S or its derivative strains for *in vivo* experiment. The overnight cultured strains were grown in LB medium at 37°C. Isopropyl β-D-1-thiogalactopyranoside (IPTG) was added to a final concentration of 1 mM at the time point when the OD_600_ reached 0.6–0.8. The culture was continuously incubated for 18 h at 20°C or for 2 h at 37°C. Afterwards cells were harvested by centrifugation, washed twice with ice-cold PBS buffer (pH 7.4) and crushed by ultrasonic cell disruptor. All subsequent procedures were performed at 4°C. 6×His-tagged protein was purified by nickel-nitrilotriacetic acid (Ni-NTA) Superflow columns (Merck) and gradient elution of imidazole. GST-tagged protein was purified by Glutathione Agarose columns. The concentration of protein was monitored by BCA method using PBS buffer as control, and the standard curve was determined by bovine serum albumin (BSA).

### *In vitro* acetylation and deacetylation of S1

Acetylation and deacetylation experiments were performed to determine whether S1 was a substrate for Pat and CobB *in vitro*. For enzymatic acetylation, the reaction was carried out at 37°C in 50 mM HEPES buffer (pH 7.5) for 1 h, with 10 μg S1, 0.2 μM Pat, and 20 μM Ac-CoA. For enzymatic deacetylation, the reaction was carried out at 37°C in 50 mM HEPES buffer (pH 8.5) for 3 h, with 10 μg S1, 1 mM MgCl_2_, 1 mM NAD^+^, and 0.2 μM CobB. After the reaction time, samples were divided into two portions. One portion was used for sodium dodecyl sulfate–polyacrylamide gel electrophoresis (SDS–PAGE) and western blot, and the other portion for other required measurements.

### Western blot analysis

Protein samples were separated by SDS–PAGE and transferred to a polyvinylidene difluoride (PVDF) membrane (Millipore). The PVDF membrane was blocked at room temperature for 2 h in BSA Blocking Buffer (CWBIO, China). After washing with Tris-Buffered Saline with Tween-20 (TBST) buffer [20 mM Tris–HCl, pH 7.6, 150 mM NaCl, and 0.1% (v/v) Tween 20], the membrane was probed with primary antibody and then incubated with secondary antibody conjugated to horseradish peroxidase (HRP). Signal detection was tested by an enhanced chemiluminescence (ECL) system (CTB, USA) according to the manufacturer. Acetyl-lysine antibody (PTM-102) was purchased from PTM BioLab (HangZhou, China). Anti-mouse IgG HRP-conjugated antibody (HS201) was purchased from Solarbio (Beijing, China).

### Identification of acetylated lysine residues by mass spectrometry

The target protein bands were cut from SDS–PAGE and washed with 50% ethanol for decolorization, and then reduced with 10 mM dithiothreitol at 56°C for 1 h. Acrylamide (55 mM) was added and alkylated for 45 min at room temperature without light. After that the protein was digested with trypsin (10 ng/μl) at 37°C for 16 h. The peptide was extracted by shaking with a solution containing 50% acetonitrile and 5% acetic acid, and then by a solution containing 75% acetonitrile and 0.1% acetic acid. The obtained peptide was desalted and then dissolved with 0.1% acetic acid solution to complete the pretreatment. The acetylation sites of proteins were determined by mass spectrometry, and the relative acetylation degree of acetylation sites were calculated by high performance liquid chromatography (HPLC) peak area.

### Expression and purification of site-specifically acetylated S1^K247Ac^

K247 site-directed acetylation protein (S1^K247Ac^) was constructed and expressed by site-specific incorporation of N^ϵ^-acetyllysine system [35, 36]. *E. coli* BL21 was transformed with plasmid pTECH-AcK3RS-PylT and pET28a-S1 K247(TAG), and grown overnight in LB supplemented with 50 μg/ml kanamycin and 50 μg/ml chloramphenicol. Three hundred milliliters of prewarmed LB was inoculated with 3 ml overnight culture, 2 mM acetyllysine (AcK) and antibiotics as required at 37°C. When OD_600_ reached 0.5, protein expression was induced by addition of 0.6 mM IPTG for 16 h at 37°C. Meanwhile, 2 mM nicotinamide (NAM) was added to inhibit the deacetylase activity and prevented it from removing the acetyl groups on the acetylated proteins. Cells were harvested by centrifugation and stored at −80°C. The purification method was described in “Overexpression and purification of proteins.”

### The anti-S1 K247Ac specific polyclonal antibody preparation

According to the sequence of protein S1, modified antigen polypeptide KVL-(acetyl)K-FDRERTRVS were designed and synthesized for animal immunity and purification. After immunization of four specific pathogen free (SPF) grade rabbits, serum was taken for ELISA and western blot to evaluate the titers and specificity of antisera. Afterwards, sufficient rabbit serum was used for Protein A and immunogen polypeptide column affinity purification. The purified antibodies were detected by enzyme-linked immunosorbent assay (ELISA), dot blot, and western blot. Nonmodified peptide NVKVLKFDRERTR was used as control. The specific polyclonal antibody customization was synthesized by PTM Biolabs lnc. (China).

### Circular dichroism spectrometry assay

The secondary structures of S1^WT^, S1^K247Ac^, S1^K247Q^, S1^K247R^, and S1^K247A^ were evaluated using circular dichroism spectrometry (Applied Photophysics, Leatherhead, United Kingdom) in the far-UV region (190–260 nm) at room temperature using a 10-mm cuvette. The proteins (0.2 mg/ml) were dissolved in a modified PBS buffer (pH 7.4) containing 1.4 M KF, 100 mM K_2_HPO_4_, and 18 mM KH_2_PO_4_. The circular dichroism spectrum scan of every sample was performed in triplicate.

### RNA hyperchromicity

The purine-rich DNA sequence (Supplementary Table S2) containing T7 promoter was synthesized as a template in the *in vitro* transcription experiment. The pyrimidine-rich RNA (Poly(rC-U)) was obtained using an *in vitro* transcription kit (Magen). For RNA hyperchromicity, 10 μl S1 (2.0 μM) was added into a reaction mixture (40 μl total volume) containing 5 mM Tris–HCl (pH 7.4), 10 mM NaCl, and 240 μg/μl Poly(rC-U) at room temperature. The absorbance was measured at 260 nm. Every sample was performed in triplicate.

### Ribosome-nascent chain complex purification

*Salmonella typhimurium* Δ*rpsA*::S1^WT^, Δ*rpsA*::S1^K247Q^, and Δ*rpsA*::S1^K247R^ were grown overnight in 5 ml of liquid LB at 37°C. After the second transfer culture to the shaking bottles and reaching OD_600_ 0.8–1.0 (exponential phase), the cells were quickly pelleted by centrifugation (6000 × *g*, 20 min, 4°C). The supernatants were removed, and the pellets were resuspended in 5 ml of prechilled Buffer B (50 mM HEPES, 500 mM KOAc, 24 mM Mg(OAc)_2_, 100 μg/ml chloramphenicol, pH 7.4) supplemented with 10 mg/ml lysozyme. After 20 min ice-bath, samples were frozen by liquid nitrogen and disrupted to powder using mechanical grinding. Cell lysates were treated with RNase-free DNase I (Thermo Scientific, USA) for 15 min on ice, and the debris was removed by centrifugation at 18 000 rpm for 15 min at 4°C. Three milliliters of supernatants were layered on 12 ml of 35% sucrose buffer, and the ribosome-associated mRNAs were pelleted after ultracentrifugation (Beckman Coulter SW 70 Ti rotor) at 42 000 rpm for 5 h at 4°C. Ribosome-associated mRNAs were isolated using the TRIzol RNA extraction reagent (Ambion, USA), according to the instructions. Genomic DNA was removed by treating with RNase-free DNase I. The 23S, 16S, and 5S rRNAs were removed using the Ribo-Zero magnetic Kit (Gram-Positive Bacteria, Epicentre, USA). Both total RNA and RNC–RNA samples were prepared from three independent experiments. Equal amount of total mRNA or RNC–RNA from each preparation was pooled, respectively, for subsequent library construction and RNA-seq. Purity, concentration and integrity of each total mRNA and RNC–RNA sample was verified by agarose gel electrophoresis and Nanodrop (Agilent 5400).

### RNC-sequencing and data analysis

The RNC–RNA libraries were generated using NEBNext^®^ mRNA Library Prep Master Mix Set for Illumina (BioLabs, USA) as directed by the manufacturer [37]. The purified libraries were sequenced on an Illumina HiSeq 2000 sequencer for 50 cycles. The reads passing the Illumina filter were mapped to *S. typhimurium* str. 14028S genome (GenBank: CP001363.1) with the criteria as followed: max read length = 60; max error = 3; indel detection = on; best position = on; min. seed length = 8; memory reduction = on. Differential expression analyses between groups were conducted using edgeR. A combined criterion of | log_2_(fold change) | ≥ 1 and a *P* < 0.05 was adopted to judge the significance of differentially translated gene (DTG) between different groups. Three biological replicates were performed for each group.

### Swim plate assay

*Salmonella typhimurium* was grown on LB solid plates overnight at 37°C. The single colonies were inoculated in LB until the exponential growth phase. The supernatant was removed by centrifugation (6000 *× g*, 10 min), and the cell pallets were resuspended in sterile water and inoculated in the middle of the semisolid medium containing 50% LB and 0.25% agar. After culture at 37°C for 8 h, the motility of Δ*rpsA*::S1^WT^, Δ*rpsA*::S1^K247Q^, and Δ*rpsA*::S1^K247R^ was compared according to the radius of bacterial movement on plate.

### Cell infection assays

HeLa cell invasion and intramacrophage replication assays were conducted to assess the virulence of *Salmonella*. For epithelial cell infection, 1 × 10^5^ cells/well of HeLa were seeded in 24-well plates and infected at multiplicity of infection (MOI) = 100 with overnight-cultured *S. typhimurium*. After 1 h of infection at 37°C with 5% CO_2_ atmosphere, cells were washed thrice with PBS and incubated in Dulbecco's Modified Eagle Medium (DMEM) containing 100 μg/ml gentamicin for 2 h to kill extracellular bacteria, then washed thrice with PBS. Infected cells were lysed by PBS containing 1% Triton-X100 and plated on LB agar after proper dilution. The number of colonies was counted to calculate invasion efficiency (percentage of the starting inoculum internalized at the end of the assay).

A total of 2 × 10^5^ mouse macrophage-like RAW264.7 cells were seeded into each well of 24-well plates in DMEM supplemented with 10% fetal bovine serum (FBS). Cells were infected at MOI = 10 with overnight-cultured *S. typhimurium*. After incubation for 1 h, extracellular bacteria were removed by extensive washing with DMEM twice and the medium were supplemented with 100 μg/ml gentamicin to culture for 2 h and then switched to medium containing 25 μg/ml gentamicin for the remainder of the experiment. The collected cells were treated as described above. The fold change of bacterial replication from 2 to 24 h post-infection in cells was measured by colony-forming unit (CFU) counting. Each assay was performed simultaneously in three separate wells, and repeated thrice. Results are presented as the mean ± SD.

### Immunoprecipitation

Immunoprecipitation (IP) of *Salmonella*-infected HeLa or RAW264.7 was performed as described followed. HeLa cells were seeded in 10-cm dishes with 3.75 × 10^6^ cells per dish, and RAW264.7 were seeded in 10-cm dishes with 9 × 10^6^ cells per dish overnight. Afterwards, cells were infected with *S. typhimurium* containing Flag-tagged S1 at MOI = 100 for HeLa and MOI = 10 for RAW264.7 for 1 h. Subsequent manipulation was according to “Cell infection assays.” Infected cells were lysed by PBS containing 1% Triton-X100 and pellets were collected by centrifugation. All subsequent procedures were performed at 4°C. The precipitates were resuspended by precooled PBS containing protease inhibitor cocktail (P1025, Beyotime Biotechnology, China) and deacetylase inhibitor cocktail (P1112, Beyotime Biotechnology, China), and crushed by ultrasonic cell disruptor. The supernatant was collected by centrifugation. The IP process was following Pierce^™^ Anti-Flag Magnetic Agarose IP Kit protocol (A36797, Thermo Fisher, USA). Obtained S1 proteins were separated by SDS–PAGE and used for Western blot analysis or mass spectrometry detection.

### Electrophoretic mobility shift assay

The 5′-biotin‐labeled probe used for the electrophoretic mobility shift assay (EMSA) was amplified by polymerase chain reaction (PCR). EMSA probe primers were listed in Supplementary Table S2. Binding reactions contained ArgR (with or without 10 mM L-Arginine), target 5′-biotin‐labeled EMSA probe, and EMSA/Gel-shift binding buffer (Beyotime Biotechnology, China) were incubated at 18°C for 30 min. After reaction, the samples were loaded with EMSA/Gel-shift loading buffer (Beyotime Biotechnology, China) and separated on a native PAGE gel in ice‐bathed 0.5 × Tris‐borate‐EDTA (TBE) at 100 V. Binding signals were detected by BeyoECL Plus (Beyotime Biotechnology, China). Specifically, the RNA–EMSA of S1 was performed using a Chemiluminescent RNA EMSA Kit (Beyotime Biotechnology, China), according to the manufacturer’s instructions.

### Quantitative real-time PCR assay

For mRNA preparation, 10 ml of stationary phase cultures were centrifuged at 4°C, and cell pellets were washed with RNase-free water. Total mRNA was prepared using RNAprep pure Cell/Bacteria Kit (Tiangen Biotech, China) and was reverse transcribed with PrimeScript^™^ RT Master Mix (Takara, Japan). Primers used in quantitative real-time PCR (qPCR)reaction were listed in Supplementary Table S2. For realtime PCR, TransStart^®^ Green qPCR SuperMix (Transgen, China) was utilized with 100 ng complementary DNA (cDNA) as templet in 20 μl of volume of qPCR reaction. The qPCR was conducted using CFX96 real-time system (Bio–Rad, USA), and the conditions were 95°C for 5 min, 40 cycles of 95°C for 5 s, and 60°C for 30 s.

### Cytokine detection

The contents of tumor necrosis factor-alpha (TNF-α), interleukin-1 beta (IL-1β), and interleukin-6 (IL-6) in the RAW264.7 supernatant 24 h.p.i were determined using a cytokine detection kit, according to the manufacturer’s instructions.

### Animal studies

Six- to eight-week-old female C57BL/6J mice were purchased from Shanghai Model Organisms Center and divided into four groups randomly. For intraperitoneal injection, cultured bacteria were diluted in saline to 1 × 10^5^ CFU/ml. Each mouse in the experimental group was injected with 100 μl of saline containing corresponding bacteria, and mice were injected with an equal volume of saline as a control.

For 72-h infection experiments, mice were euthanized 72 h after injection. CFU in the livers and spleens were determined by plating proper serial dilutions of livers or spleens suspended in sterile 0.1% Triton X-100 PBS on agar plates. Resulting quantities were normalized to liver or spleen weight.

For the *S. typhimurium* infection survival assay, weight loss and survival of mice were monitored starting just before infection, and mice were euthanized when they reached 80% baseline weight, appeared hunched or moribund or exhibited a visibly distended abdomen, whichever occurred first. Death was not used as an end-point.

### Ethics statement

All animal experiment procedures were approved by East China University of Science and Technology, and were performed in strict accordance with the Guidelines for Care and Use of Laboratory Animals [ECUST-21038]. The experiments and all efforts were made to minimize suffering.

### Statistical analysis

All experimental samples were carried out in triplicate, and the data were expressed as mean ± standard deviation (SD), while statistical significance significant differences were analyzed with *t*-test or one-way analysis of variance (ANOVA) analysis. *P< 0.05, **P< 0.01, ***P< 0.001, ****P< 0.0001, ns, *P*> 0.05. GraphPad Prism 8.0 was used to generate graphs and conduct data analysis. ImageJ was utilized to analyze the grayscale of western blot.

## Results

### K247 of S1 is acetylated dynamically in simulated infection process of *S. typhimurium*

**Figure 1.**
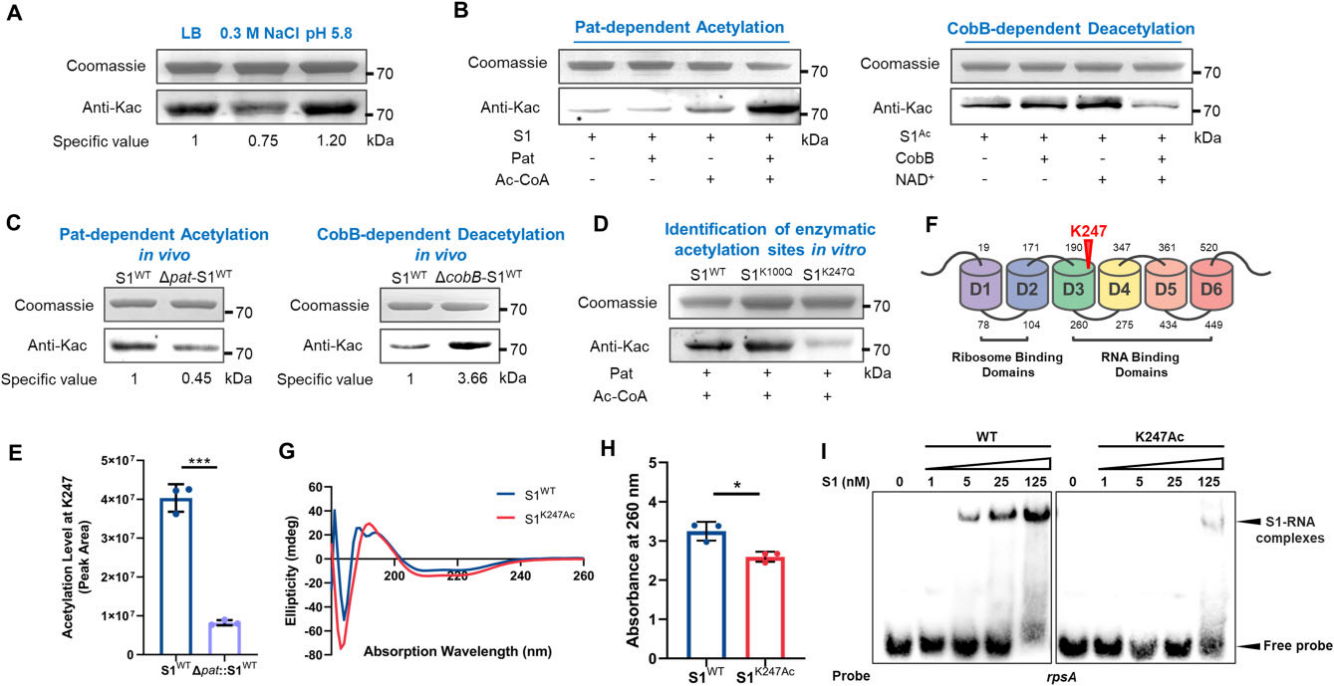
Pat-dependent acetylation of S1 at K247 regulated its structure and function. (**A**) Acetylation degree and of S1 protein under different simulated physiological conditions (LB medium alone or supplied with 0.3 M NaCl or pH 5.8, respectively). Acetylation levels were determined by western blot analysis with the pan anti-acetyllysine antibody (anti-Kac), and the Coomassie was used as a loading control. (**B**) S1 can be enzymatically acetylated by Pat. *In vitro* experiment incubated S1 (0.2 μg/μl) with or without 0.2 μM Pat and 20 μM Ac-CoA. S1 can be enzymatically deacetylated by CobB. *In vitro* experiment incubated the acetylated S1^Ac^ (0.2 μg/μl) from Δ*cobB* background with or without 1 mM NAM adenine dinucleotide (NAD^+^) and 0.2 μM CobB. (**C**) S1 can be enzymatically acetylated and deacetylated *in vivo*. 6×His-tagged S1 from WT, Δ*pat*, or Δ*cobB S. typhimurium* 14028S was expressed and purified at 37°C. (**D**) The effect of Pat on S1^WT^ and variants (S1^K100Q^ and S1^K247Q^). WT S1 and variants were incubated with Pat and Ac-CoA at 37°C for 2 h. (**E**). The semi-quantitative analysis of the acetylation degree at K247 of S1 from WT and Δ*pat* background *S. typhimurium* 14028S. 6×His-tagged S1 was purified and analyzed with LC/MS/MS after trypsin digestion. Three replicates were made for each sample; ***P < 0.001, Student’s *t-*test. (**F**) Structural domain organization of S1 is shown in differently colored boxes. The triangle shows the position of acetylated site lysine 247. (**G**) Circular dichroism spectra of S1^WT^ and site-specifically lysine acetylated S1^K247Ac^. Three replicates were made for each sample. (**H**) Effect of S1^WT^ and site-specifically lysine acetylated S1^K247Ac^ on the induction of hyperchromicity in poly(rC-U). RNA hyperchromicity of S1 on poly(rC-U) was measured at 260 nm. Three replicates were made for each sample; *P < 0.05, *t-*test. (**I**) K247Ac impairs the binding ability of S1 to specific S1-dependent mRNA (*rpsA* enhancer). The *rpsA* enhancer is the 11-bp upstream of SD sequence of *rpsA* in *S*.Tm. REMSA was used to test the binding of S1^WT^ and S1^K247Ac^ to 5′-biotin-labeled-specific mRNA probe. In each group, the lower band is the free probe, and the position marked by the arrow above is the S1-RNA binding band. The assay was done in duplicate three independent measurements.

In our investigation into the acetylation dynamics of S1 during *S. typhimurium* infection, we established two distinct simulated conditions to mimic bacterial invasion (0.3 M NaCl) and intracellular replication within macrophages (at pH 5.8), respectively [38]. Notably, the acetylation status of S1 exhibited a contrasting pattern under these conditions: a decrease in 0.3 M NaCl and an increase at pH 5.8 compared to standard LB media alone (Fig. 1A). Through enzymatic assays, we demonstrated the reversible acetylation of S1 by the enzymes Pat and CobB *in vitro* (Fig. 1B). Subsequently, we validated the *in vivo* acetylation status of S1 by expressing and purifying S1 in *S. typhimurium* strains lacking either *pat* (Δ*pat*) or *cobB* (Δ*cobB*). As depicted in Fig. 1C, the acetylation level of S1 in the Δ*pat* strain was significantly lower than that of the WT S1, exhibiting a reduction of ∼55%. Conversely, the Δ*cobB* strain displayed a markedly elevated acetylation level, ∼3.7 times higher than the WT *S. typhimurium* strain. These combined *in vivo* and *in vitro* findings underscored the precise and complicated modulation of S1 by the Pat/CobB enzymatic system. Furthermore, through mass spectrometry analysis under pH 5.8 conditions, we identified two specific acetylated sites on the S1 protein: K100 and K247 (Supplementary Fig. S1). To assess the impact of Pat on these two identified residues, we generated substitution mutations to create the S1^K100Q^ and S1^K247Q^ variants. Our results revealed that both S1^WT^ and S1^K100Q^ were susceptible to acetylation by Pat *in vitro*, whereas S1^K247Q^ showed minimal acetylation, indicating that K247 serves as a Pat-specific catalytic site rather than K100. Semi-quantitative analysis using HPLC further confirmed the enzymatic acetylation of K247 by Pat both *in vitro* and *in vivo* (Fig. 1D and E, and Supplementary Fig. S2A and B).

In the majority of gram-negative organisms, the ribosomal protein S1 typically comprises six repeats of the S1 domain, with K247 localized within domain D3, a region crucial for mRNA binding (Fig. 1F). Through circular dichroism assays, we observed that S1^K247Ac^ induced subtle alterations in the secondary structure of S1, characterized by a modest increase in helical content and a slight decrease in random coil structures, ∼9% and 5%, respectively (Fig. 1G; Supplementary Fig. S3A and Supplementary Table S3). The consistent secondary structure data were observed in S1 derivative mutants K247Q and K247R (Supplementary Fig. S3B and Supplementary Table S3). Furthermore, comparative analyses revealed that S1^K247Ac^ disrupted the RNA-binding capacity of S1, as evidenced by both RNA hyperchromicity assays and RNA-electrophoretic mobility shift assay (REMSA) with S1-specific binding mRNA (Fig. 1H and I and Supplementary Fig. S4A). Notably, analogous results were obtained with both K247Q and K247Ac variants (Supplementary Fig. S4B and Supplementary Table S3), suggesting that K247Q could serve as a suitable mimic for the acetylation state in subsequent *in vivo* investigations. These findings underscore the intricate interplay between acetylation at position K247 and the structural and functional dynamics of ribosomal protein S1, shedding light on its role in mRNA recognition and processing in gram-negative pathogens.

### S1^K247^ and S1^K247Ac^ recruit different specific mRNAs

**Figure 2.**
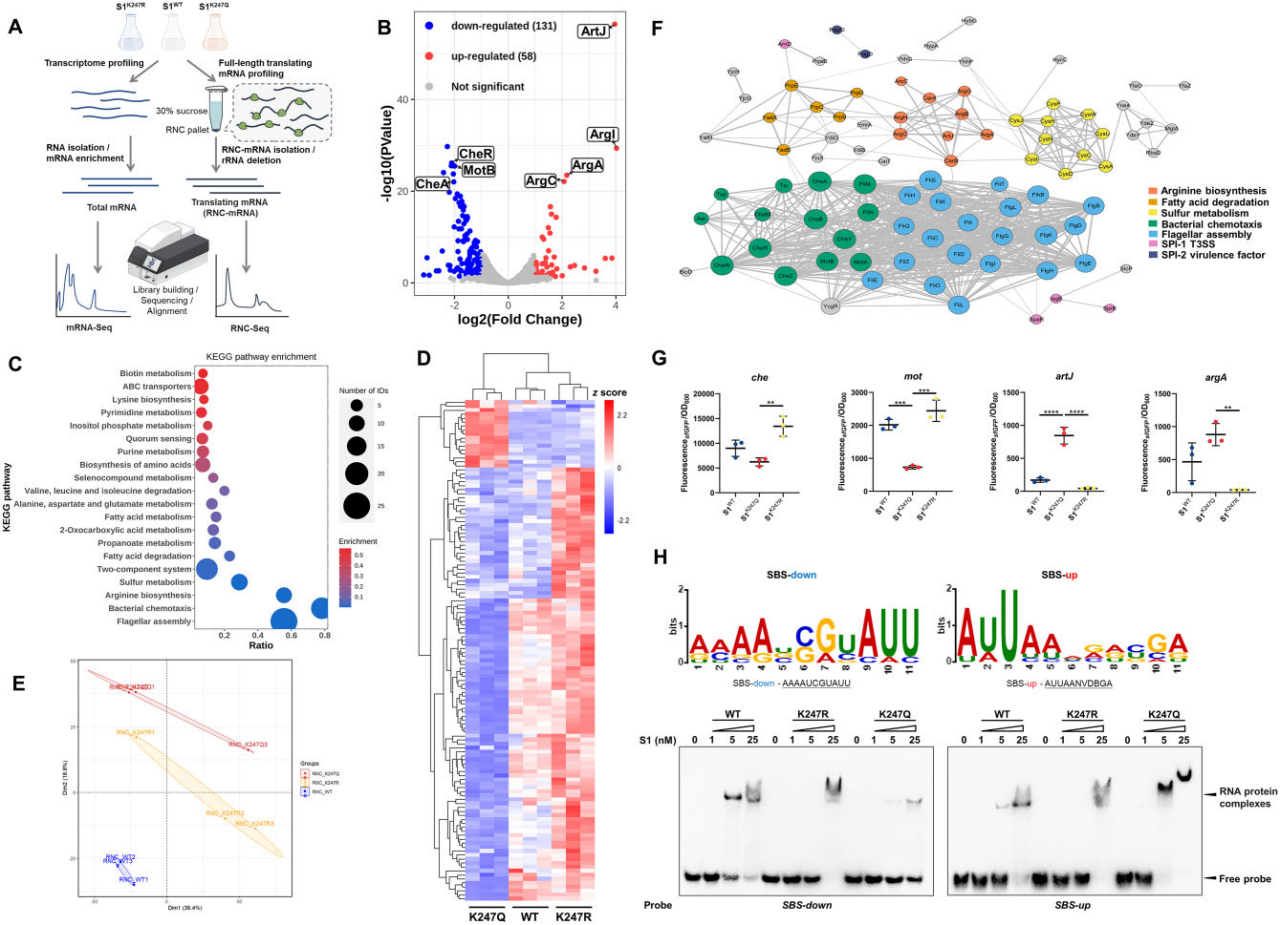
S1 heterogeneity caused by dynamic state of acetylation at lysine 247 recruits specific mRNAs to translate. (**A**) The workflow of the ribosome-nascent chain complex sequence (RNC-Seq) and mRNA-Seq of simulated acetylated/deacetylated S1 (K247Q/K247R) or WT S1-containing ribosomes. (**B**) Volcano plots about thresholds of differentially expressed genes (DEGs) in K247Q/K247R- RNC-Seq (*P*< 0.05, log_2_FC>1). (**C**) Bubble plot of KEGG pathway enrichment analysis of differential genes. (**D**) Cluster map of DEGs in S1 K247Q/WT/K247R- RNC-Seq (*P*< 0.05, log_2_FC>1). The RPKM value of DEGs under different experimental conditions was used as the expression level, and the log_10_(RPKM + 1) value was used for hierarchical clustering analysis. Regions with different colors represent differential expression levels and different clustering grouping information. (**E**) Principal component analysis of each experimental sample. Three biological replicates were generated for RNC-Seq libraries. (**F**) Network-based cluster assay of DEGs in K247Q/K247R- RNC-Seq and their intertwined functional classes according to KEGG pathway classification. (**G**) Relative fluorescence unit (RFU) of SBS-down (*che*/*mot*) or SBS-up (*artJ*/*argA*)-guiding sfGFP in Δ*rpsA*::S1^WT^, Δ*rpsA*::S1^K247Q^ or Δ*rpsA*::S1^K247R^ strains. RFU is equal to fluorescence/OD_600_.Three independent single colony were made for each sample. Data represent mean ± s.d. **P< 0.01, ***P< 0.001, ****P< 0.0001, one-way ANOVA analysis. (**H**) The SBS plays a crucial role in K247-acetylated S1-mediated translational regulation. SBS is defined as the 11 nucleotides of mRNA immediately upstream of the Shine–Dalgarno sequence of target mRNAs. Analysis of conserved SBS of the up- or down-regulated genes regulated by K247Q/K247R. The top 20 transcripts in up- or down-regulated samples were analyzed using MEME. REMSA was used to test the binding of S1 WT, K247R, and K247Q to 5′-biotin-labeled RNA containing SBS-up (AUUAANVDBGA) and RNA containing SBS-down (AAAAUCGUAUU). The assay was done in duplicate three independent measurements.

To investigate the differential recruitment of mRNA transcripts associated with the acetylation status of K247, we conducted ribosome-nascent chain complex sequencing (RNC-Seq) as depicted in Fig. 2A. We transformed the constitutive plasmid to express S1^WT^, S1^K247Q^, and S1^K247R^ in WT *S. typhimurium*, respectively. Then, the endogenous *rpsA* gene was deleted, and three mutant strains were successfully obtained (Supplementary Fig. S5A). Subsequently, mRNA fragments undergoing translation within ribosomes harboring S1^K247Q^ and S1^K247R^ were selectively isolated and sequenced post ribosome removal, enabling precise quantification of the recruited mRNA species. Notably, S1^WT^ served as a stringent quality control reference throughout the experimental procedures, ensuring robust experimental standards (Supplementary Fig. S5B–D). As shown in Fig. 2B, there were two subsets of translation dynamics: an up-regulated set comprising 58 genes and a down-regulated set comprising 131 genes in ribosomes bearing S1^K247Q^ compared to those with S1^K247R^. The detailed information of differentially expressed proteins enriched by RNC-Seq was presented in Supplementary Table S4. Subsequently, KEGG pathway functional enrichment analysis showed the differential genes, which predominantly encompassed flagellar assembly, bacterial chemotaxis, arginine biosynthesis, sulfur metabolism, and the TCS (Fig. 2C). A comprehensive delineation of the all three subsets of the translatome was present through a heat map of the differential gene cluster analysis and principal component analysis. (Fig. 2D and E). As expected, the data for the WT strain are positioned between those for the K247Q and K247R mutants, with a closer resemblance to the K247R mutant. Previous findings (Fig. 1A and Supplementary Fig. S1) have demonstrated that acetylation at position K247 occurs under conditions of pH 5.8, and that the abundance of K247 acetylation in the WT strain is minimal when grown in LB medium alone (Supplementary Fig. S3A). Consequently, the WT strain predominantly exists in a nonacylated state. Moreover, network-based cluster analysis provided further insights into the interplay and mRNA preference selectively captured by S1^K247Q^ and S1^K247R^ (Fig. 2F). Particularly noteworthy is the discernment that S1^K247R^ preferentially translates proteins associated with bacterial chemotaxis, flagellum assembly, and sulfur metabolism, while S1^K247Q^ displays a bias towards arginine biosynthesis and SPI-2 virulence factors.

Previous reference has reported that protein S1 interacted with ∼11 nucleotides of mRNA immediately upstream of the Shine–Dalgarno sequence [31], we focused on this 11-bp length motif designated as the S1-binding sequence (SBS). To further demonstrate that the selectively recruitment of mRNA results in differential protein synthesis level, we assessed the intensity of a fluorescence reporter (superfolder green fluorescent protein, sfGFP) guided by the SBS. The SBS of downregulated gene clusters *che* and *mot*, as well as the upregulated genes *artJ* and *argA*, were chosen as representative examples. Based on fluorescence intensity measurements, we confirmed that the distinct acetylation status of K247 caused significant differences in protein synthesis level (Fig. 2G). Additionally, to explore the effect of 11-bp SBS on translational regulation, we compared the characteristics of SBS sequences of up- or down-regulation of RNC-Seq data using MEME (Multiple EM for Motif Elicitation). Analysis of the SBS sequences revealed the presence of conserved motifs, underscoring the critical role of the SBS in acetylated S1-mediated translational regulation (Fig. 2H). Collectively, our findings underscore the dynamic recruitment of specific mRNA subsets facilitated by Pat-mediated acetylation of S1 at position K247, thereby empowering *S. typhimurium* to cope with fluctuations in external environmental cues.

### S1^K247^ is beneficial for invading epithelial cells

**Figure 3.**
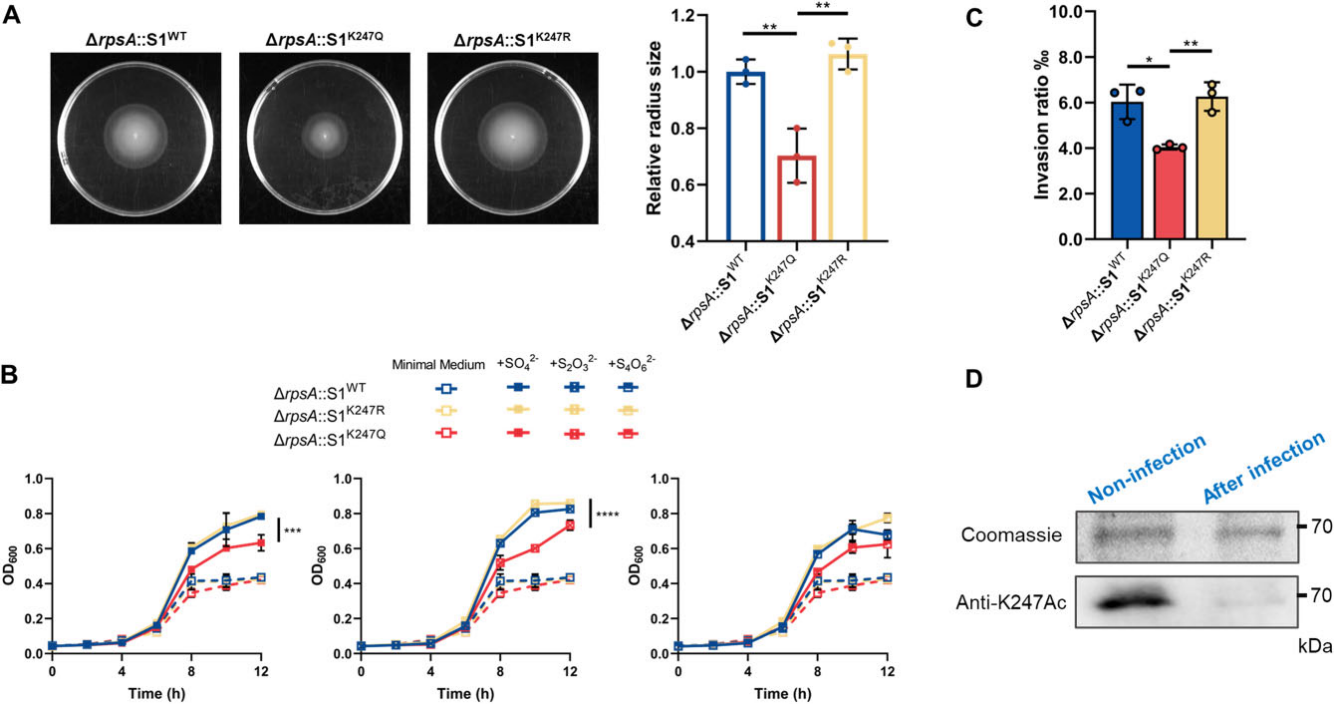
S1^K247^ is beneficial for invading epithelial cells. (**A**) Swim plate assay of S1^WT^, S1^K247Q^, and S1^K247R^ on semisolid medium. Migration capacity was measured by diameter and normalized compared with S1. The assay was done in three independent measurements, with spotting on three plates in each case, and one representative image is shown for each strain. Data represent mean ± s.d. **P< 0.01, one-way ANOVA analysis. (**B**) Growth curve of the *S. typhimurium* Δ*rpsA* strains complemented with WT S1 or K247 mutants in M9 minimal medium or supplied with 2 mM Na_2_SO_4_ (+SO_4_^2-^), 1 mM Na_2_S_2_O_3_ (+S_2_O_3_^2−^), and 0.5 mM Na_2_S_2_O_3_ (+S_4_O_6_^2−^), respectively. Graphed points represent the means of three independent measurements. Data represent mean ± s.d. ***P < 0.001, ****P < 0.0001, one-way ANOVA analysis. (**C**) Invasion abilities of WT S1 (Δ*rpsA*::S1) and variants Δ*rpsA*::S1^K247Q^ and Δ*rpsA*::S1^K247R^ strains into HeLa cells. HeLa cells were infected at an MOI of 10. Cells lysates were plated on agar plates, and bacterial colonies were calculated the invasion efficiency. The invasion abilities were measured in three independent experiments. Data represent mean ± s.d. *P< 0.05, **P< 0.01, one-way ANOVA analysis. (**D**) Acetylation level of S1 K247 in HeLa. After 2 h treatment, the intracellular bacteria were harvested for IP assay. Flag-tagged S1 proteins were immunoprecipitated by the anti-flag antibody. The acetylation level of K247 was determined by the anti-S1 K247Ac site-specific antibody with western blot assay. Coomassie was used as loading control. Three independent measurements were made for each sample.

Our investigation revealed a significant enrichment and upregulation of mRNAs associated with flagellar assembly, bacterial chemotaxis, sulfur metabolism, and SPI-1 T3SS in the S1^K247R^ group. Subsequent traditional swim plate assays were carried out to indicate a nearly two-fold increase in swarm diameter for K247R and WT compared to K247Q (Fig. 3A). To further investigate the growth advantages of *S. typhimurium* strains under conditions of external sulfate, thiosulfate, and tetrathionate, we supplemented nutrient-limiting media with 2 mM sulfate, 1 mM thiosulfate, and 0.5 mM tetrathionate, respectively. Our results demonstrated that K247R exhibited growth patterns similar to WT and outperformed K247Q significantly (*P* < 0.001) in the presence of sulfate and thiosulfate (Fig. 3B). These suggests that *S. typhimurium* carrying the S1^K247R^ mutation may establish a competitive advantage by forming a niche during invasion of host intestinal epithelial cells through sulfate matabolism. Subsequent infection of HeLa cells with S1^WT^, S1^K247R^, and S1^K247Q^ mutants of *S. typhimurium* revealed that both WT and K247R displayed substantially higher invasion ratios compared to K247Q (Fig. 3C, *P* < 0.01). Additionally, examination of the acetylation state of S1 at K247 during infection revealed a remarkable decrease in acetylation levels when WT *S*. *typhimurium* infected HeLa cells, as evidenced by western blot analysis using an anti-K247Ac site-specific antibody (Fig. 3D). Semi-quantitative mass spectrometry data also confirmed that K247 existed in a deacylated state during HeLa cell invasion (Supplementary Fig. S6A and B). In conclusion, our findings suggest that the acetylation status of S1 at K247 undergoes a transition to a nonacetylated state during infection, allowing for the selective recruitment of virulence genes associated with flagellar assembly, bacterial chemotaxis, sulfur metabolism, and SPI-1 T3SS for translation.

### S1^K247Ac^ promotes the replication and survival within macrophages

**Figure 4.**
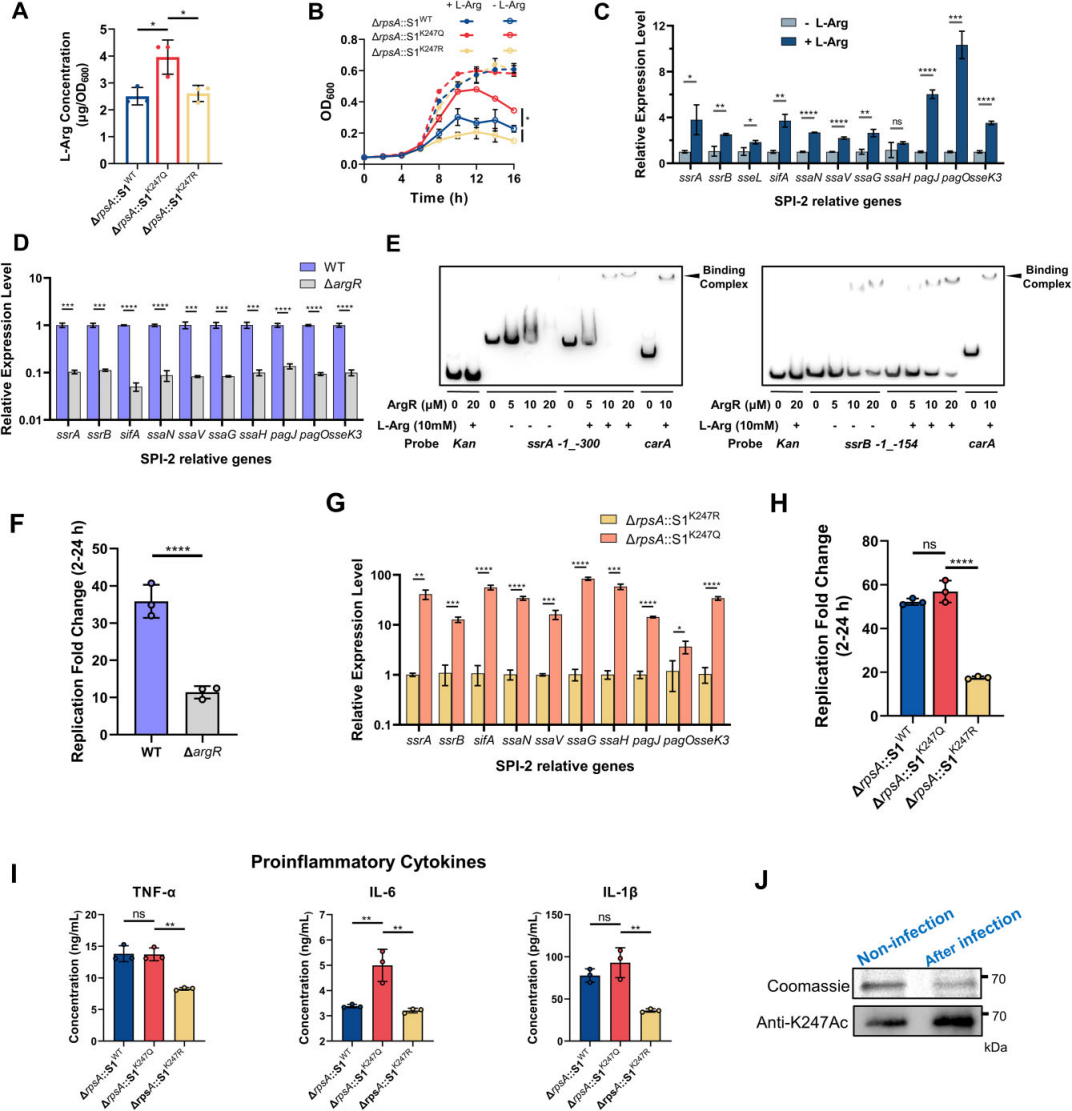
S1^K247Ac^ promotes the replication and survival of *S. typhimurium* in macrophages. (**A**) Determination of bacterial intracellular L-arginine content by ELISA. Each group contained three single colony. (**B**) Growth curve of the *S. typhimurium* Δ*rpsA* strains complemented with S1 WT or K247 mutants in N limiting medium (-L-Arg) or supplied with L-Arg (+L-Arg). Each group contained three single colony. (**C**) The transcription levels of SPI-2 relative genes of *S. typhimurium* WT in N limiting medium with or without L-Arg. Each three independent samples were determined by qPCR with the methods of 2^−ΔΔCt^. (**D**) The transcription levels of virulence genes of *S. typhimurium* WT and Δ*argR* strain in N limiting medium supplied with 10 mM L-Arg. Each three independent samples were determined by qPCR with the methods of 2^−ΔΔCt^. (**E**) L-Arg increases the binding ability of ArgR to *ssrA*/*ssrB* regulatory region. EMSA was used to test the binding of ArgR at indicated concentrations to 5′-biotin-labeled corresponding promoter probes. EMSA of ArgR with kanamycin promoter probe (negative control), *carA* promoter probe (positive control), and segments of the *ssrA*/*ssrB* regulatory region numbered from the proximal transcriptional start site. In each group, the lower band is the free probe, and the position marked by the arrow above is the protein–DNA binding band. (**F**) The replication of *S. typhimurium* WT and Δ*argR* in RAW264.7 cells. RAW264.7 were infected at MOI of 100. The fold change from 2 to 24 h.p.i was calculated to present survival and replication ability. The survival and replication abilities were measured in three independent experiments. (**G**) The relative transcription levels of virulence genes of variant strains (Δ*rpsA*::S1^K247Q^ and Δ*rpsA*::S1^K247R^). Each three independent samples were determined by qPCR with the methods of 2^−ΔΔCt^. (**H**) The replication of WT S1 (Δ*rpsA*::S1^WT^) and variant strains (Δ*rpsA*::S1^K247Q^ and Δ*rpsA*::S1^K247R^) within RAW264.7 cells. RAW264.7 were infected at MOI of 100. The survival and replication abilities were measured in three independent experiments. (**I**) Cytokines detection by ELISA. RAW264.7 cells were infected with WT S1 (Δ*rpsA*::S1) variant strains (Δ*rpsA*::S1^K247Q^ and Δ*rpsA*::S1^K247R^) at MOI of 100 for 24 h. Cell culture supernatants were collected to detect the amounts of several cytokines after infection. (**J**) Acetylation level of S1 K247 in RAW264.7 macrophages. After 18 h treatment, the intracellular bacteria were harvested for IP assay. Flag-tagged S1 proteins were immunoprecipitated by the anti-flag antibody. The acetylation level of K247 was determined by the anti-S1 K247Ac site-specific antibody with western blot assay. Coomassie was used as loading control. Data represent mean ± s.e.m. *P< 0.05, **P< 0.01, ***P< 0.001, ****P< 0.0001, ns, no significance, one-way ANOVA analysis.

The RNC-Seq translatome analysis revealed a significant upregulation of the arginine biosynthesis pathway in the K247Q group (Fig. 2B and D). Previous studies by Zelia *et al.* have highlighted the dual role of arginine as a crucial modulator of host immune responses to pathogens and as a direct influencer of bacterial virulence [39]. In our study, we assessed the intracellular arginine concentrations in *S. typhimurium* strains carrying S1^WT^, S1^K247Q^, and S1^K247R^, revealing that the arginine levels in K247Q were notably higher than those in WT and K247R (Fig. 4A). Furthermore, under nitrogen-restricted conditions without L-arginine, K247Q exhibited superior growth compared to WT and K247R. However, upon supplementation with 10 mM L-arginine, the growth rates of K247R and WT were restored to levels comparable to that of K247Q (Fig. 4B). Of note, we observed a significant increase in the transcription levels of SPI-2 T3SS genes in WT *S. typhimurium* upon the addition of 10 mM L-arginine (Fig. 4C). Collectively, these observations underscore the critical role of intracellular L-arginine for *S. typhimurium* survival in restrictive environments.

ArgR, a transcription factor involved in regulation of arginine biosynthesis and metabolism in prokaryotes, assumes a critical function in perceiving and responding to L-arginine, thereby orchestrating the induction of virulence gene expression in Enterohemorrhagic *Escherichia coli* (EHEC) [39]. Notably, our investigations unveiled a parallel role for ArgR in the modulation of SPI-2 T3SS transcriptional levels within *S. typhimurium* upon genetic deletion of *argR* (Fig. 4D). Furthermore, our EMSA corroborated the direct binding of ArgR to the promoter regions of *ssrA* and *ssrB*, with a discernible enhancement in binding affinity observed in the presence of L-arginine (Fig. 4E). Significantly, at the cellular level, we observed impaired intracellular replication of the *ΔargR* strain within macrophages (Fig. 4F). These findings demonstrate intracellular L-arginine is important to facilitate ArgR binding to the promoter regions of *ssrA* and *ssrB*, thereby culminating in the upregulation of SPI-2 T3SS gene expression and heightened cytotoxicity in macrophages.

In line with the heightened intracellular L-arginine content in the K247Q group, the expression level of SPI-2 T3SS genes in the K247Q strain was significantly elevated compared to that in K247R (Fig. 4G). To substantiate the role of K247 acetylation in *S. typhimurium*’s survival within macrophages and its capacity to induce systemic infection, RAW264.7 cells were infected with S1^WT^, S1^K247R^, and S1^K247Q^ mutants in *ΔrpsA S. typhimurium*. Remarkably, K247Q and WT strains exhibited a substantial ∼58-fold increase in proliferation within macrophages from 2 to 24 h post-infection (h.p.i), whereas the K247R variant displayed an obvious decrease in intracellular viability, showing only around a 15-fold proliferation (Fig. 4H). Consistent with the cell infection assay results, K247Q demonstrated the highest levels of proinflammatory cytokines TNF-α, IL-6, and IL-1β, particularly when compared to those produced by K247R at 24 h.p.i (Fig. 4I). To verify the acetylation status of K247 within macrophages, Flag-tagged S1 proteins were subjected to IP after 18 h of infection. Subsequently, western blot analysis was performed using an anti-S1 K247Ac site-specific antibody. As illustrated in Fig. 4J, a pronounced elevation in the acetylation level of K247 was detected in intracellular bacteria compared to the control. This finding underscores the dynamic nature of protein acetylation as a crucial regulatory mechanism employed by *S. typhimurium* to adapt and thrive within the intracellular environment of macrophages.

### S1^K247Ac^ enhances bacterial virulence during murine infection

**Figure 5.**
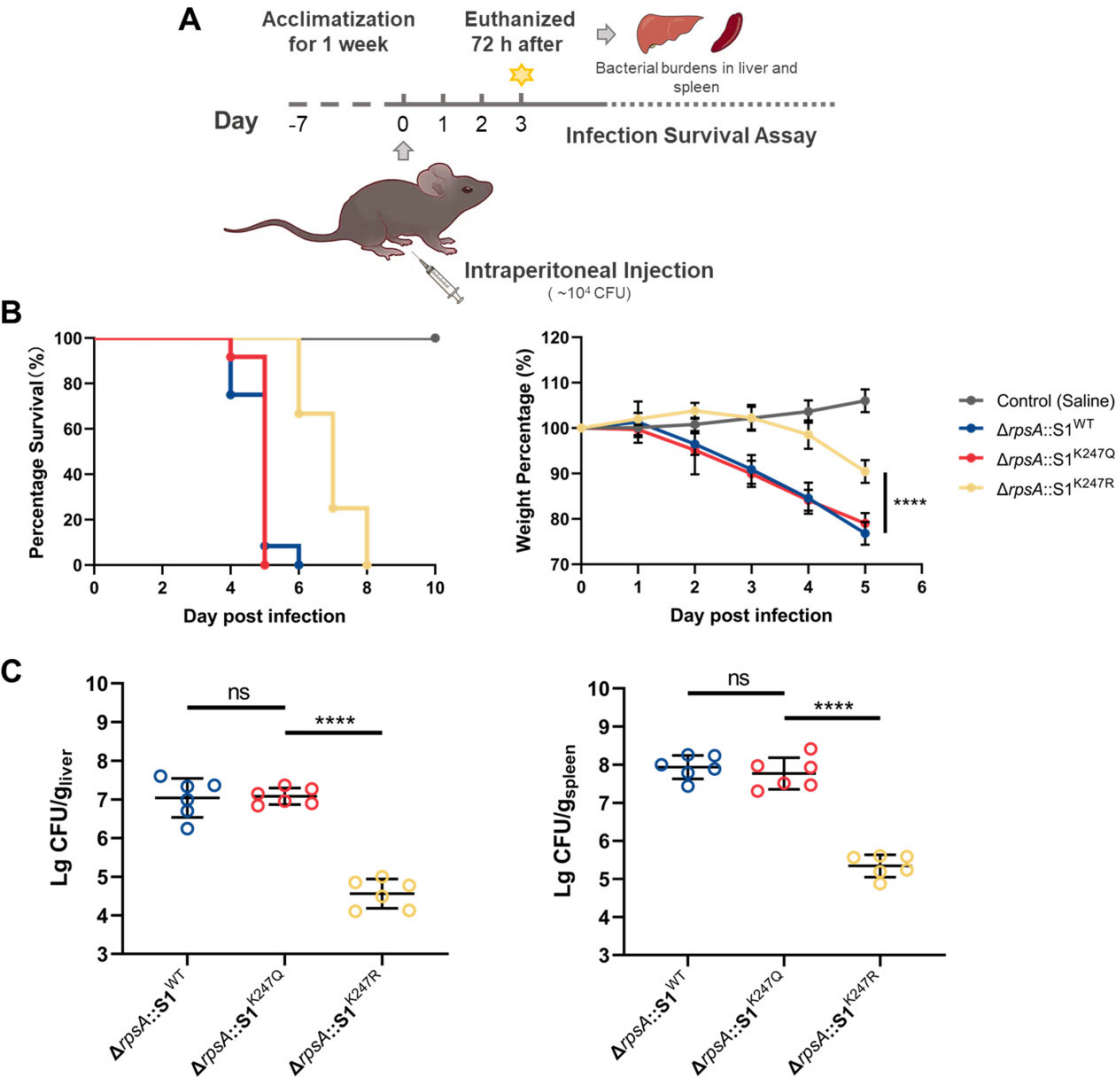
S1^K247Ac^ enhances bacterial virulence during murine infection. (**A**) Workflow of the intraperitoneal injection of *S. typhimurium* Δ*rpsA* strains and variants for toxicology test. (**B**) Survival and weight rates of mice infected by intraperitoneal injection. Eight-week-old female C57BL/6 mice were administered intraperitoneally by 1 × 10^4^ bacteria in 100 μl of saline. Control mice were given 100 μl of saline. The number of live mice was counted twice a day. (**C**) Bacterial burdens in liver and spleen. The livers and spleens were harvested 72 h after intraperitoneal injection, and bacterial colonies (CFU) were counted to analyze the number of bacteria in these organs (mean ± SD, *n* = 6, individual dots represent individual mice); ****P< 0.0001, ns, no significance, one-way ANOVA analysis.

To assess the impact of K247-acetylated S1 on the virulence of *S. typhimurium* in mice, we conducted intraperitoneal injections of three strains, *ΔrpsA*::S1^WT^, *ΔrpsA*::S1^K247Q^, and *ΔrpsA*::S1^K247R^ into female C57BL/6 mice (Fig. 5A). Each group of 10 mice received an intraperitoneal injection containing 1 × 10^4^ CFU of bacteria or saline as a control. Monitoring over a 10-day period revealed distinct outcomes. Mice infected with Δ*rpsA*::S1^K247Q^ and Δ*rpsA*::S1^WT^ exhibited rapid mortality starting on day 4, characterized by fur ruffling and marasmus, with all succumbing by days 5 and 6, respectively. In contrast, the Δ*rpsA*::S1^K247R^ group displayed reduced toxicity, demonstrating a 2-day extension in survival time (Fig. 5B). Consistent with the survival data, the weight profiles of the infected mice further underscored the virulence disparities. Mice infected with K247Q and WT strains experienced a precipitous decline in weight from the first day post-infection, reaching 80% of their original weight by day 5. Conversely, mice infected with the K247R strain initially exhibited a modest increase in weight for the first two days, followed by a gradual decline, stabilizing at ∼90% of their original weight by day 5 (Fig. 5B). These observations highlight the critical role of acetylated S1 residue K247 in modulating the virulence of *S. typhimurium* in a murine model, emphasizing its significance in the pathogenesis of systemic infection.

Furthermore, the systemic dissemination of *S. typhimurium* can result in bloodstream infection and subsequent dissemination to vital organs such as the spleen and liver. To evaluate its virulence in systemic infection, we quantified bacterial loads in these organs. Infection with K247Q or WT strains led to significantly elevated bacterial burdens in both the spleen and liver relative to those infected with the K247R strain (Fig. 5C). Notably, comparable bacterial loads were observed in the spleen and liver between the WT and K247Q cohorts, underscoring the indispensable role of K247 site acetylation in augmenting virulence during persistent systemic infection. This observation underscores the critical significance of K247 acetylation in augmenting the pathogenicity of *S. typhimurium* during systemic dissemination, thus elucidating a fundamental mechanism underpinning its virulence in the context of systemic infection.

## Discussion

**Figure 6.**
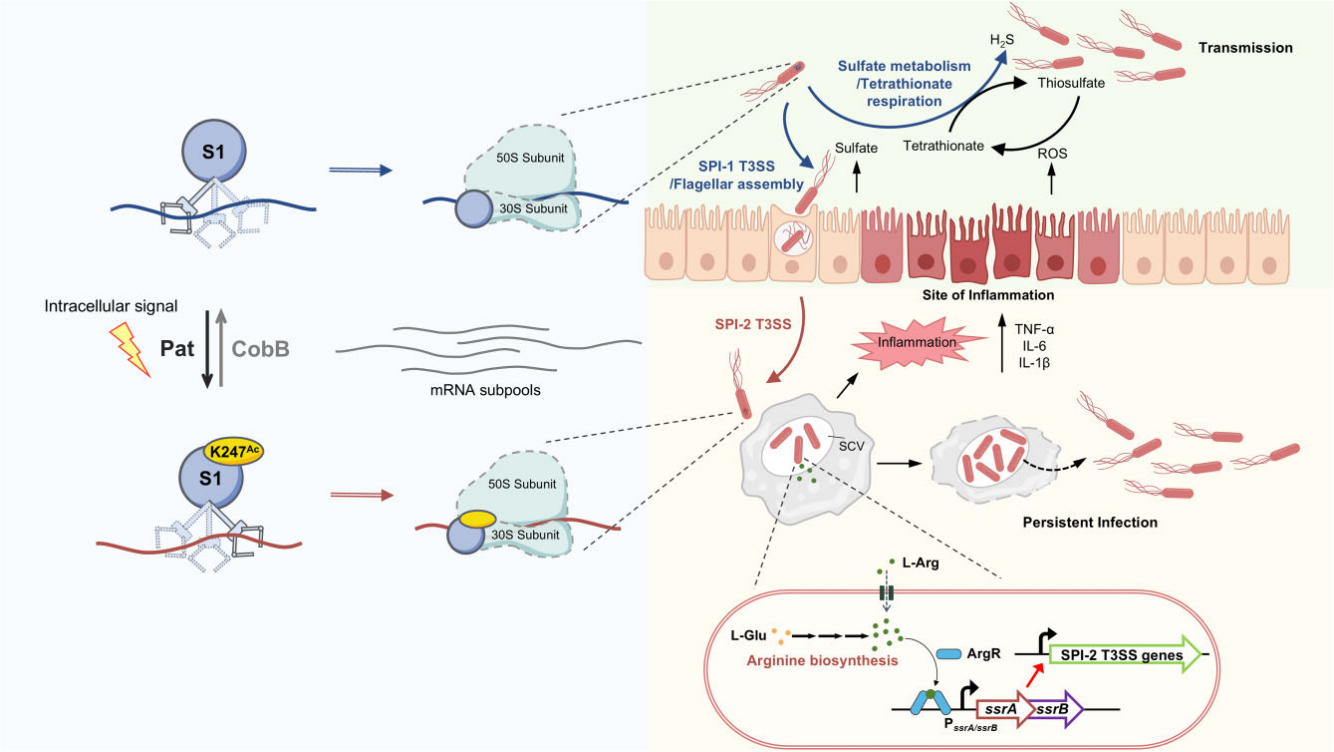
Pat-mediated enzymatic acetylation of S1 dynamically regulates the selective recruitment of mRNAs to regulate the virulence of *Salmonella* at different stages of infection.

Here, we posit a novel mechanism suggesting that *S. typhimurium* employs acetylation of the lysine residue at position 247 of the S1 protein to regulate translation reprogramming in response to host stress during infection. This allows the bacterium to quickly adapt to adverse conditions encountered throughout different stages of infection (Fig. 6).

Invasion of HeLa cells by *S. typhimurium* requires maintenance of the deacetylated K247 on S1 (Fig. 3D, and Supplementary Fig. S6A and B). In this state, S1 selectively recruits mRNAs associated with flagellar assembly, bacterial chemotaxis, sulfur metabolism, and SPI-1 T3SS (Fig. 2D and E). These factors are crucial for *S. typhimurium* to effectively invade the intestinal epithelium. *Salmonella typhimurium* takes advantage of the inflammatory environment in the intestine, where the accumulation of sulfate and its derivatives provides a growth advantage [40, 41]. Additionally, the motility conferred by flagella enables *S. typhimurium* to navigate through the mucus layer lining the intestinal epithelium and facilitate invasion [42]. Subsequently, the SPI-1 gene cluster orchestrates the injection of virulence factors into epithelial cells, culminating in the completion of the invasion process [43, 44]. Overall, the transition of K247 on S1 to a nonacetylated state orchestrates the coordinated action of relevant proteins, thereby accelerating the invasion process.

While within macrophages, an acidic environment can cause the acetyltransferase Pat to promote acetylation at K247 (Fig. 4J and Supplementary Fig. S7), leading to an increase of intracellular concentrations of L-arginine (Fig. 4A), which is a vital molecule for the adaptation in macrophages [45, 46]. L-arginine serves as a stabilizer in the active form of ArgR hexamer and increases its DNA affinity by allosteric activation [47]. These hexamers bind to the transcriptional regulatory sequence of *ssrA/B* to activate the transcription and expression of SsrA and SsrB, which leading to the upregulation of SPI-2 T3SS expression and concomitant augmentation of virulence within macrophages. Furthermore, murine infection experiments also demonstrate the K247Q strain exhibits increased virulence compared to the K247R strain (Fig. 5B and C). These collective findings underscore the K247 acetylation in modulating arginine-related mRNA capture, intracellular L-arginine accumulation, and subsequent virulence enhancement in *S. typhimurium*, shedding light on potential targets for therapeutic intervention in combating *Salmonella* infections.

Recent studies have increasingly highlighted the key role of ribosomal protein S1 in translation regulation and its versatility. The diversity of S1-binding sequence (SBS) is thought to underlie this regulatory capacity [48]. In particular, S1 ability to bind diverse mRNA sequences enables it to promote translation initiation [49], modulate translation efficiency [50], and potentially influence translation elongation and termination [48]. In this study, we demonstrate that the acetylation state of K247 in S1 selectivity recruits different mRNAs, thereby promoting pathogenic virulence. The third OB-fold domain of S1 plays a crucial role at the interface between RNA polymerase to the ribosome, with key residues in this domain (Y205, F208, H219, and R254) shown to be essential for interaction with Qβ RNA [51]. Given that K247 resides within D3 domain and near the mRNA interaction sites, we hypothesize that acetylation at K247 may alter the protein’s conformation by neutralizing the positive charge on lysine and introducing a bulkier side chain, which could affect its ability to bind mRNAs. Furthermore, the D3 domain’s role in connecting RNA polymerase to the ribosome may coordinate transcription and translation, thereby influencing the expression of virulence genes during infection. To explore these possibilities, future experiments should focus on structural studies, such as cryogenic electron microscopy (cryo-EM) or nuclear magnetic resonance spectroscopy (NMR), to visualize the effects of K247 acetylation on S1 conformation and interactions, as well as functional assays to assess its impact on transcription–translation coupling.

In addition, we performed a conserved sequence analysis to assess the potential prevalence of acetylation regulation at K247 across other strains, particularly in pathogenic organisms, such as *Acinetobacter baumannii*,*Klebsiella pneumoniae*,*Shigella sonnei*,*Pseudomonas aeruginosa*,*Pseudomonas putida*, and *Vibrio cholerae* (Supplementary Fig. S8). The results revealed that the K247 residue and its adjacent domain are highly conserved. These findings suggest that the dynamic acetylation of K247 as a regulatory mechanism for virulence may be widely applicable to other pathogenic microorganisms. This could offer a novel framework for the prevention and treatment of infections, as well as for the development of new therapeutic strategies in the future.

In this study, we proposed that K247 of the S1 protein serves as an exquisite dual-mode switch, skilled at facilitating the precise capture of specific mRNAs by modulating its acetylation status. This sophisticated mechanism affords the pathogen a nimble capability to swiftly and efficiently adapt to the environmental pressures encountered.

## Supplementary Material

gkaf252_Supplemental_File

## Data Availability

Sequences of translating mRNAs from RNC-Seq and mRNA-Seq are available on NCBI Dataset, https://www.ncbi.nlm.nih.gov/geo/query/acc.cgi?acc=GSE262202. All data generated or analyzed during this study are included in this published article or in Supplementary Data S1. The uncropped and unedited blot/gel images are available in Supplementary Data S2. The ID numbers in GenBank: *pat* (11756548), *cobB* (11756861), *rpsA* (11757724), *argR* (11758540), *ssrA* (11756932), *ssrB* (11755263), *sseL* (11755969), *sifA* (11755117), *ssaN* (11758325), *ssaV* (11756938), *ssaG* (11755271), *ssaH* (11758322), *pagJ* (11756882), *pagO* (11757523), and*sseK3* (11757239). The protein ID numbers in UniProt: Pat (A0A0F6B583), CobB (A0A0F6B059), S1 (A0A0F6AZD0), and ArgR (A0A0F6B7F2).
