# Supplementary material for: Reversible acetylation of ribosomal protein S1 serves as a smart switch for Salmonella to rapidly adapt to host stress
Source: Nucleic Acids Res. 2025 Apr 1;53(6):gkaf252. doi: 10.1093/nar/gkaf252 (PMC11959540; doi:10.1093/nar/gkaf252)
Supplement: gkaf252_Supplemental_File [file gkaf252_supplemental_file.pdf]

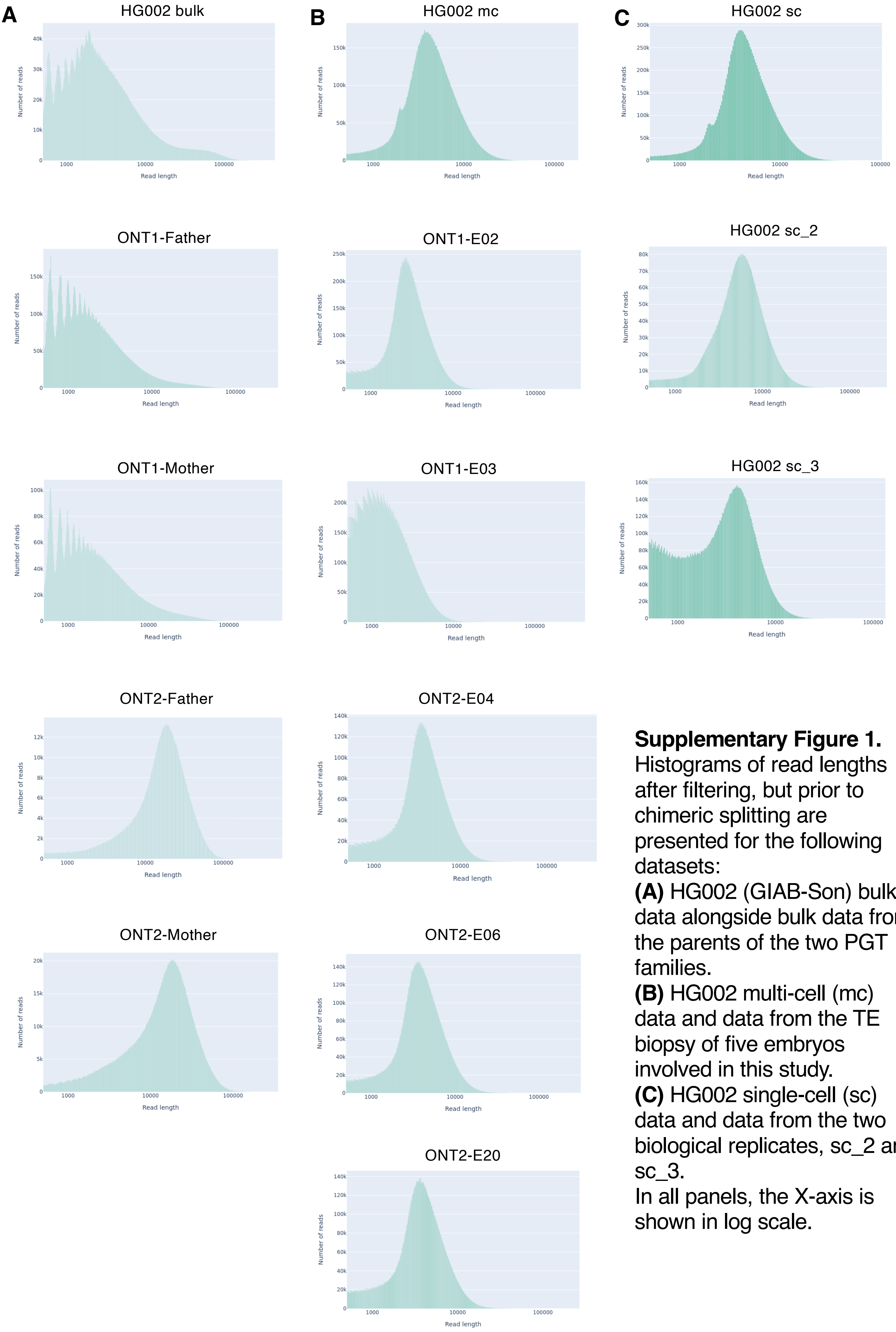

Supplementary Figure 1. Histograms of read lengths after filtering, but prior to chimeric splitting are presented for the following datasets:
(A) HG002 (GIAB-Son) bulk data alongside bulk data from the parents of the two PGT families.
(B) HG002 multi-cell (mc) data and data from the TE biopsy of five embryos involved in this study.
(C) HG002 single-cell (sc) data and data from the two biological replicates, sc_2 and sc_3.
In all panels, the X-axis is shown in log scale.

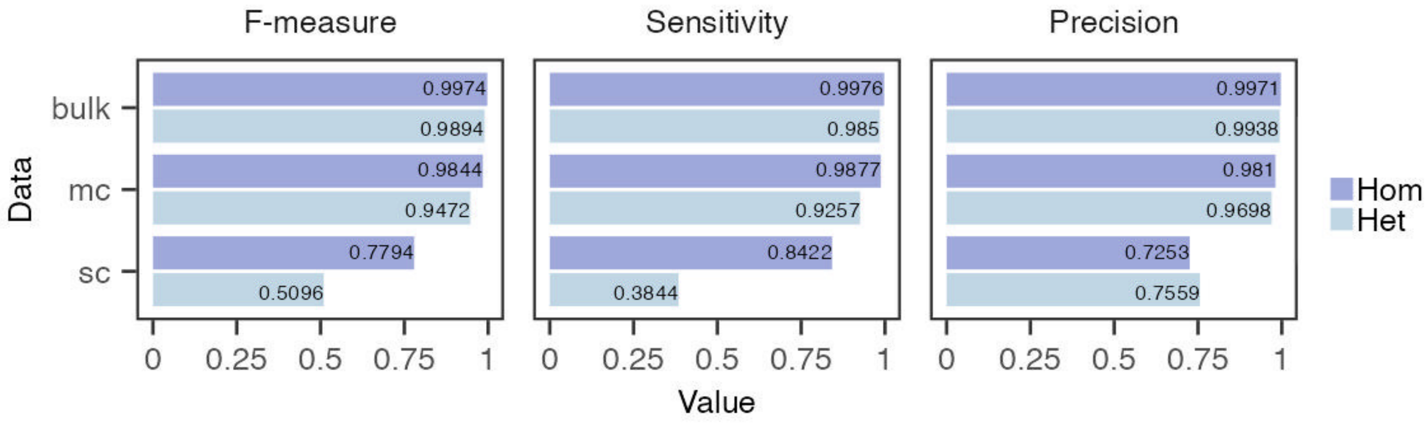

Supplementary Figure 2. Heterozygous (Het) and homozygous (Hom) SNV calling performance for lrWGS data from single-cell (sc), multi-cell (mc), and bulk samples of the offspring.

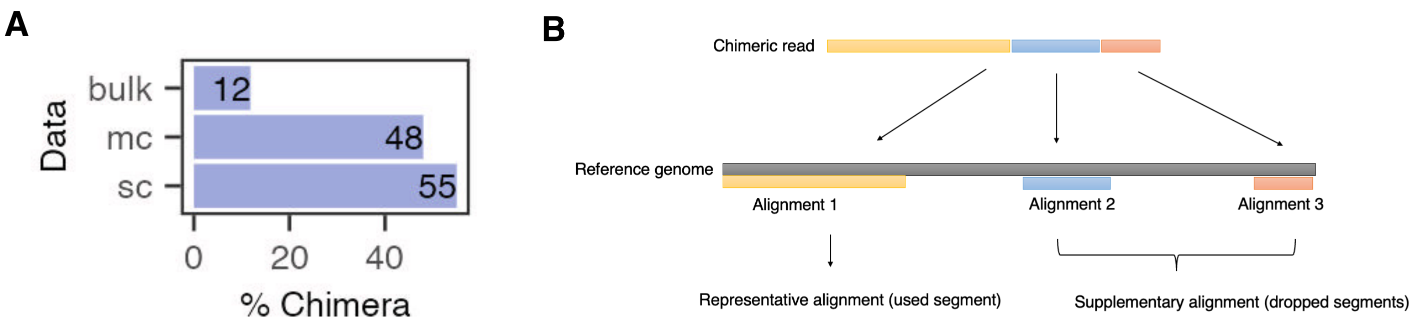

C

Data	Type	Mean	Median	N50
mc	Before split	6,307	5,200	7,532
mc	After split	2,415	1,901	3,570
sc	Before split	6,151	5,152	7,070
sc	After split	2,255	1,815	3,284

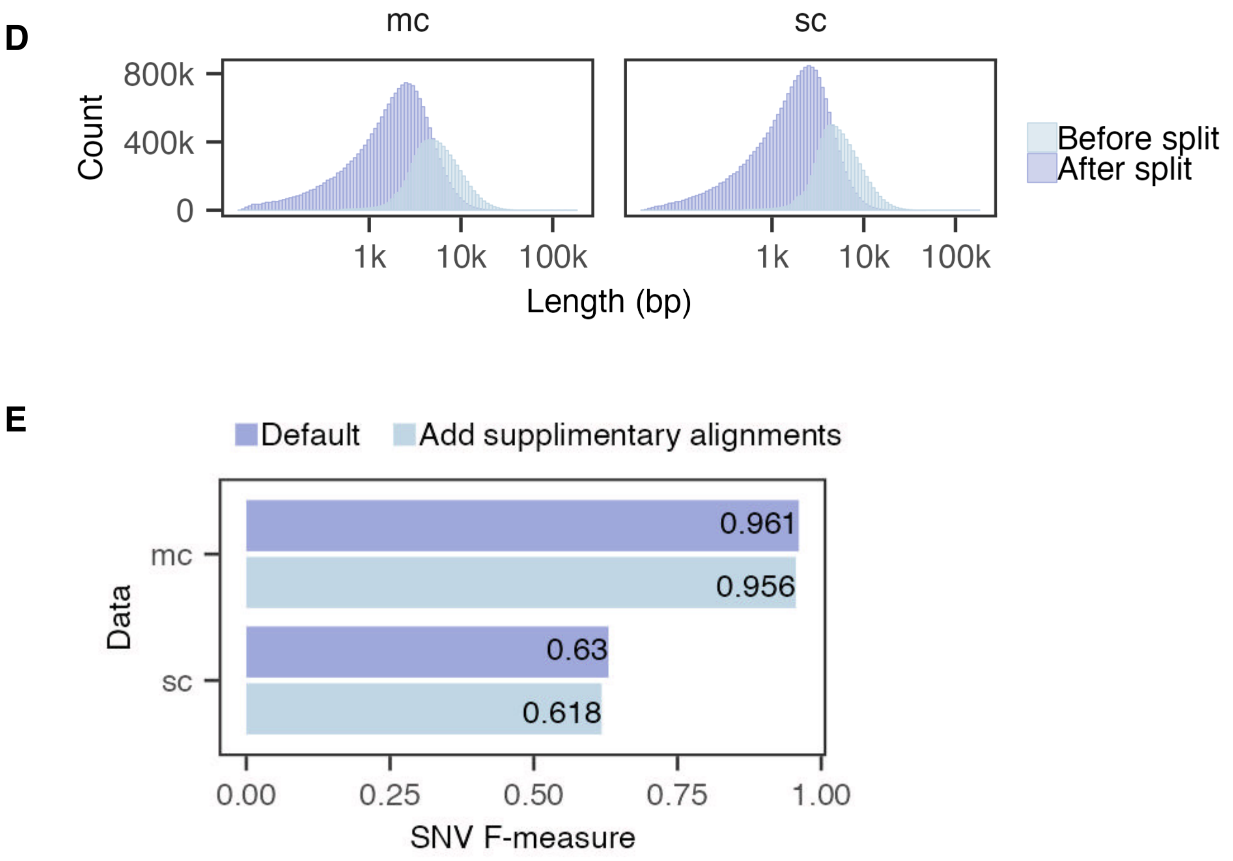

Supplementary Figure 3. Supplementary alignments did not improve SNV calling performance. **(A)** Percentage of chimeric reads in bulk, multi-cell (mc) and single-cell (sc) lrWGS data of the offspring. **(B)** Schematic representation of chimeric reads and supplementary alignments. **(C)** Length statistics for chimeric reads (before splitting) and their derived segments (after splitting) in sc and mc data. **(D)** Length distribution histograms of chimeric reads (before splitting) and their derived segments (after splitting) in sc and mc data. The X-axis is shown in log scale. **(E)** Comparison of the default SNV calling F-measures with those obtained after incorporating supplementary alignments for SNV calling.

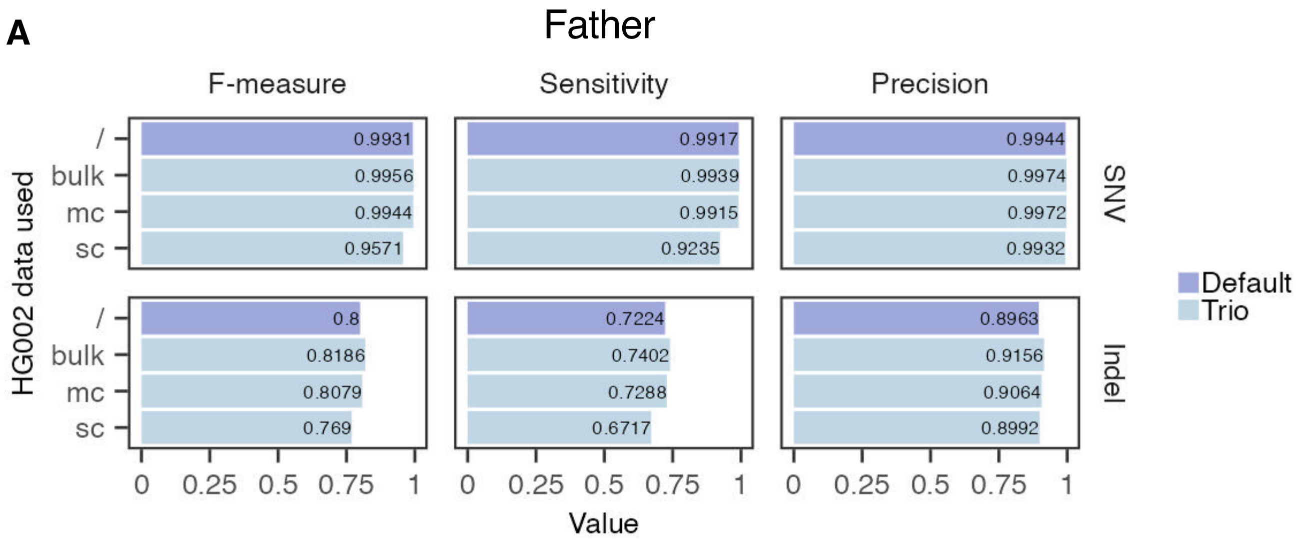

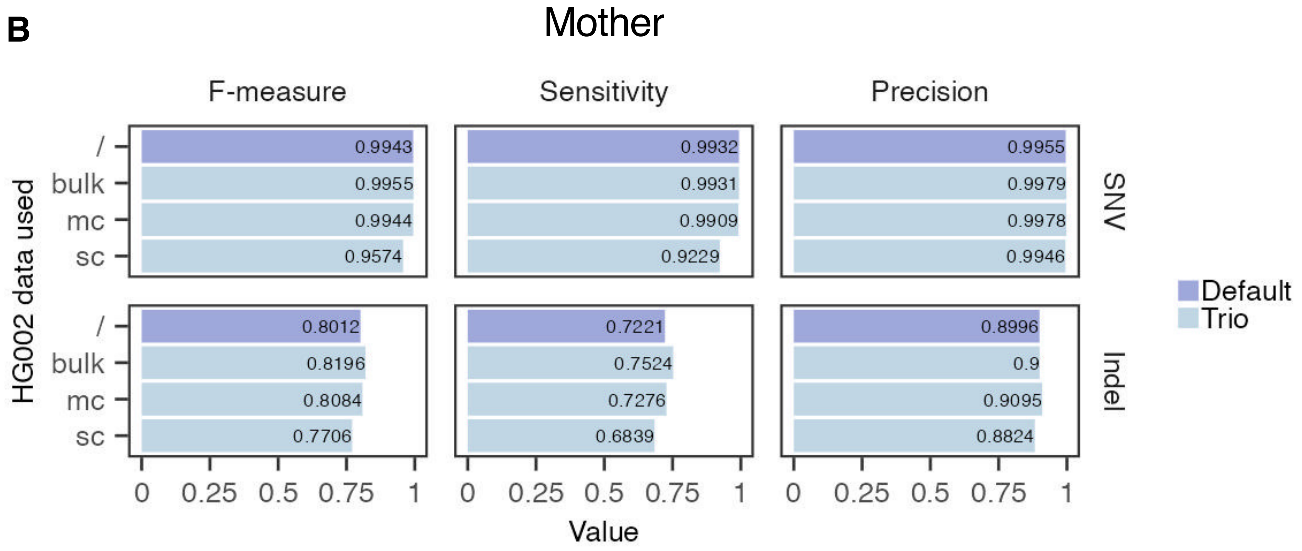

Supplementary Figure 4. Small variant (SNV and indel) calling performance obtained form default and trio variant calling for **(A)** the father and (**B**) the mother. On the y-axis, 'bulk,' 'mc,' and 'sc' indicate that bulk, multi-cell (mc), or single-cell (sc) data from the offspring were used for trio variant calling along with parental data. The "/" indicates that only paternal or maternal data were utilized for variant calling.

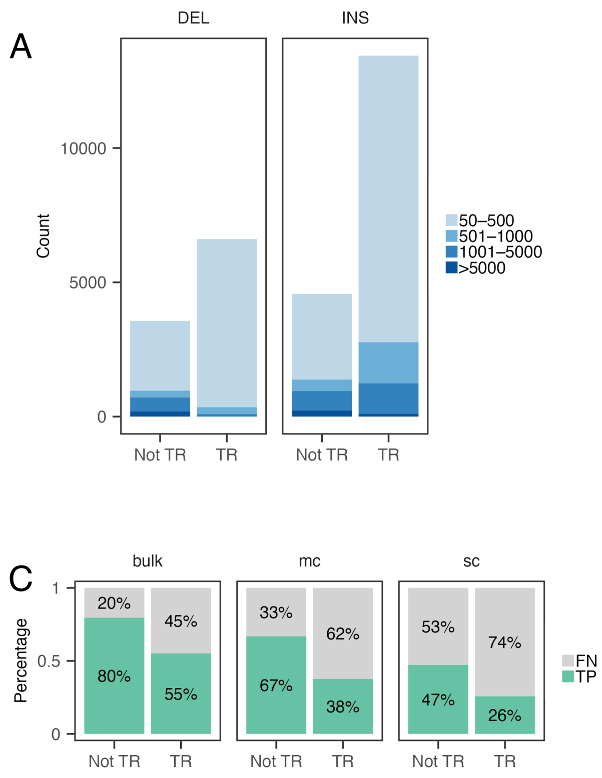

Supplementary Figure 5. SV calling performance. **(A)** Number of deletions (DEL) and insertions (INS) in the benchmark set located within tandem repeat regions (TR) and outside tandem repeat regions (Not TR). Variants of different length ranges are shown in varying shades of blue, as indicated in the legend (bp). **(B)** SV calling performance metrics (F-measure, sensitivity, and precision) for bulk, multi-cell (mc), and single-cell (sc) data of HG002. **(C)** Percentage of SVs in the benchmark set, located either outside or within tandem repeat regions, that were correctly identified (TP, true positives) or missed (FN, false negatives) for bulk, mc, and sc data.

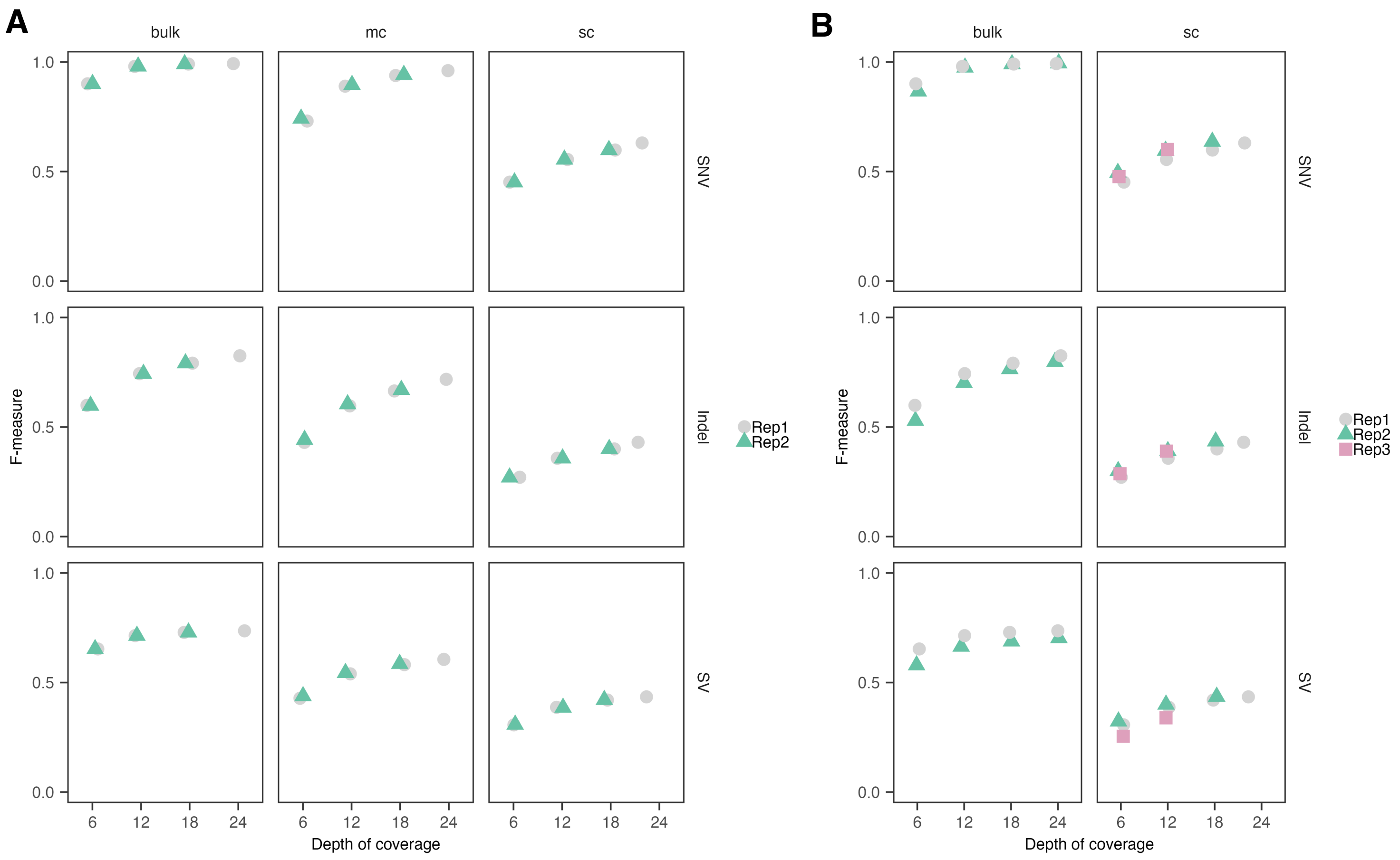

Supplementary Figure 6. Concordance of variant calling F-measure for (A) technical and (B) biological replicates of different sample types at varying depths of coverage. In (A), technical replicates were generated by randomly downsampling the bulk, multi-cell (mc), and single-cell (sc) data of HG002 to specific coverage levels (6x, 12x, and 18x), with each level performed twice (Rep1 and Rep2). In (B), the biological replicate for the bulk sample of the offspring (Rep1) was obtained from the same source as the parental bulk data, referred to as Rep2, and downsampled to 6x, 12x, 18x and 24x. Two additional biological replicates for the single-cell sample (Rep1) were created: sc_2 (Rep2, 19x) and sc_3 (Rep3 ,14x). Rep2 was downsampled to 6x, 12x, and 18x, while Rep3 was downsampled to 6x and 12x.

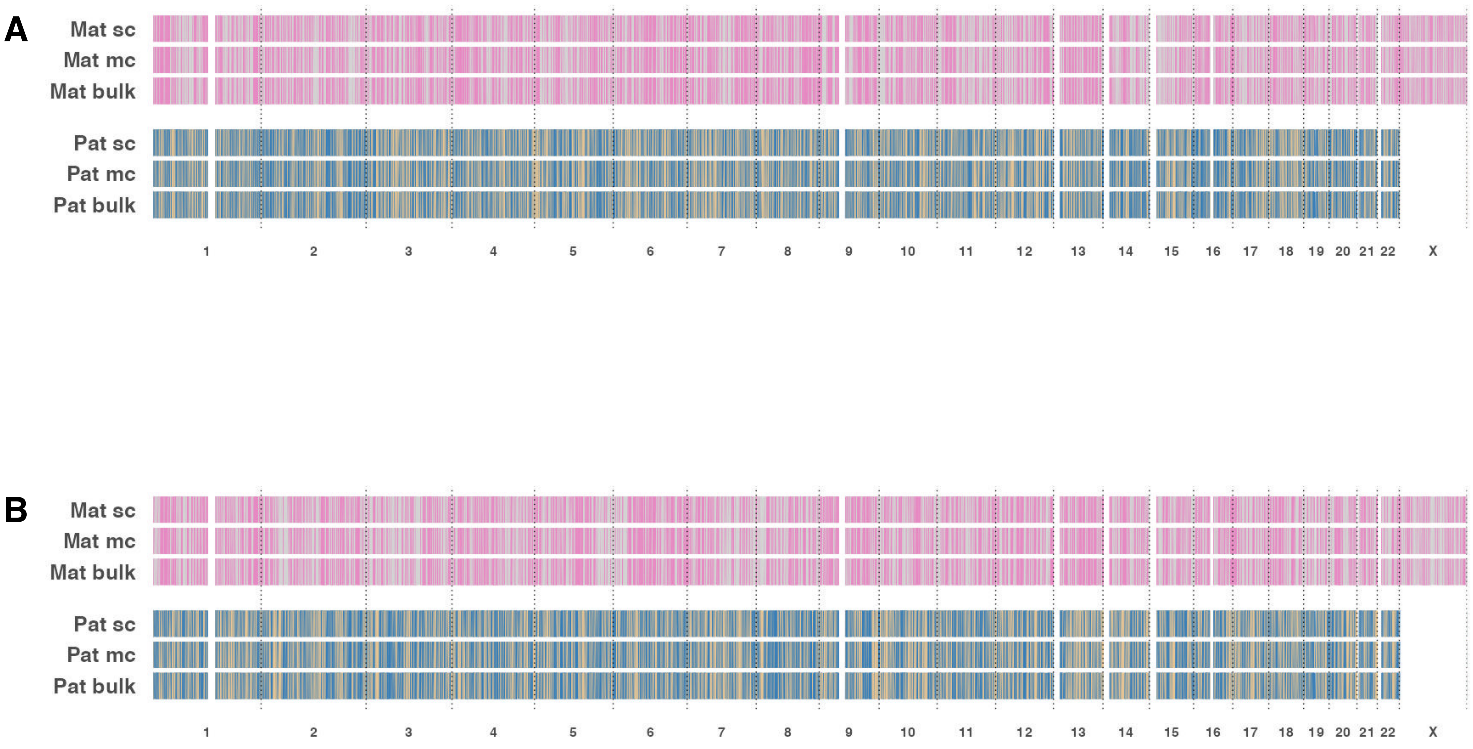

Supplementary Figure 7. Consistency of transmitted parental haplotypes inferred from the offspring's bulk, multi-cell (mc) and single-cell (sc) data phasing results when phasing **(A)** only SNVs **(B)** SNVs and indels. For each panel, vertical colored lines indicate inferred paternal (pat) or maternal (mat) haplotypes for specific regions. Different parental haplotypes are distinguished by different colors.

A

<table>
<tr><th colspan="3">Trio genotype combinations for informative loci</th><th rowspan="2">Phasing method</th></tr>
<tr><th>Father</th><th>Mother</th><th>Offspring</th></tr>
<tr><td>0|1 or 1|0</td><td>0|1 or 1|0</td><td>0|1 or 1|0</td><td>Read only</td></tr>
<tr><td>0/0 or 1/1</td><td>0|1 or 1|0</td><td>0|1 or 1|0</td><td>Genetic or read</td></tr>
<tr><td>0|1 or 1|0</td><td>0/0 or 1/1</td><td>0|1 or 1|0</td><td>Genetic or read</td></tr>
<tr><td>0/0</td><td>1/1</td><td>0|1 or 1|0</td><td>Genetic or read</td></tr>
<tr><td>1/1</td><td>0/0</td><td>0|1 or 1|0</td><td>Genetic or read</td></tr>
</table>

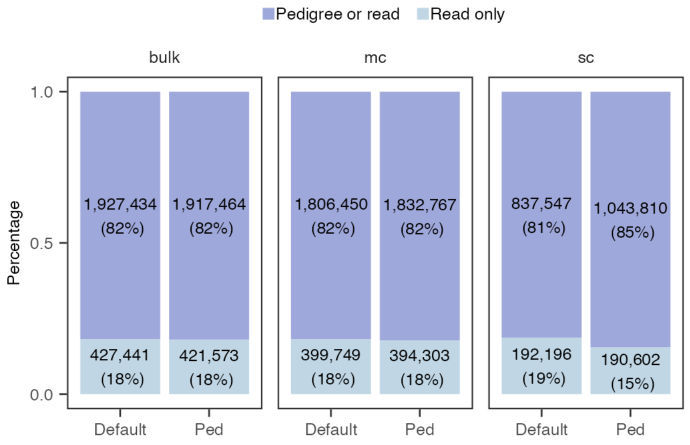

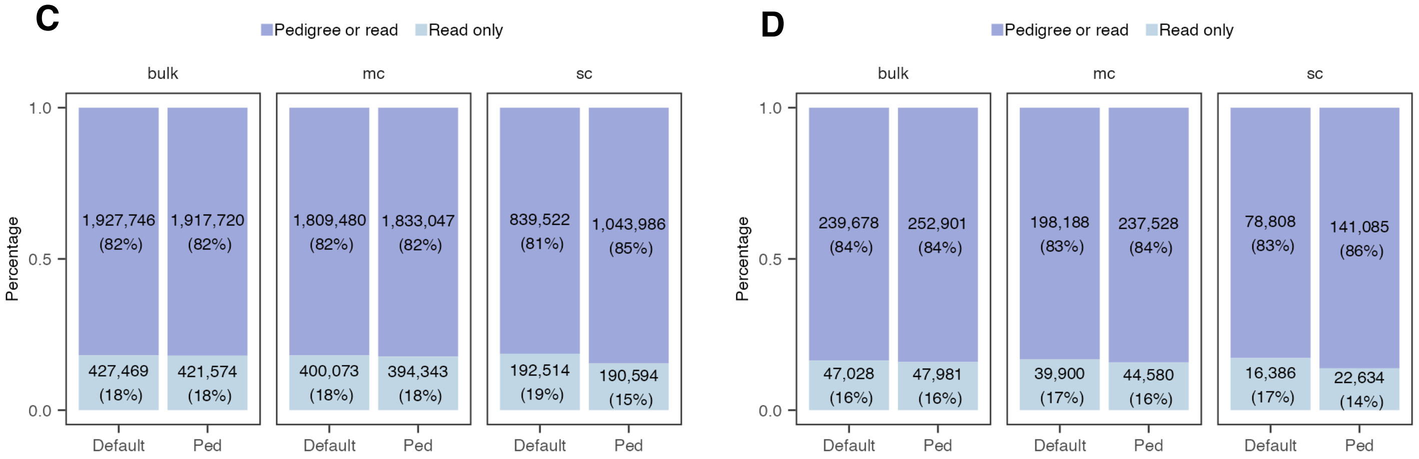

Supplementary Figure 8. Proportion of informative loci that can only be phased by long reads in default or pedigree (Ped) phasing results of bulk, multi-cell (mc) and single-cell (sc) data. **(A)** Trio genotype combinations for loci retained as informative loci for haplotype comparison. The “Phasing method” column indicates method(s) that could be used to phase variants loci of the offspring. Read only: variant loci could only be phased through read-based phasing. Genetic or read: variant loci could be phased by either genetic phasing or read-based phasing. **(B-D)** Informative **(B)** SNVs from SNV phasing results, **(C)** SNVs from SNV and indel phasing results, and **(D)** indels from SNV and indel phasing results retained for haplotype comparison, categorized by phasing method(s) that could be used to phase the variant loci.

Supplementary Figure 9. SNP array-based comprehensive PGT results for **(A)** ONT1-E02, **(B)** ONT1-E03, **(C)** ONT2-E04, **(D)** ONT2-E06 and **(E)** ONT2-E20. For each embryo, the first panel displays genome-wide maternal and paternal haplarithm plots (Mat-BAF and Pat-BAF tracks) along with a copy number plot (logR track) for chromosomes 1 to X. For family ONT1, haplotyping was performed with paternal grandparents, and thus, only the paternal haplarithm is shown. For family ONT2, haplotyping was performed with a sibling, allowing both maternal and paternal haplarithms to be displayed. Aneuploidies are identified via the logR track, with chromosomal gains marked by red rectangles and chromosomal losses by green rectangles. Combining this information with the paternal and maternal haplarithms helps determine both the parental and mechanistic origins of the aneuploidies. For disomic chromosomes, a distance of 0.5 between the red and blue lines for paternal and maternal haplarithms is expected. Pure trisomy is indicated by a distance of 0.67 in one parent's haplarithm and 0.33 in the other parent's haplarithm. The parent with a distance of 0.33 contributed two chromosomes: when the red and blue lines are at 0 and 0.33 or at 0.67 and 1, this indicates two identical haplotypes from that parent, whereas a distance of 0.33 and 0.67 signifies two different haplotypes from that parent. This information helps identify the parental and mechanistic origin of the trisomy. For mosaic aneuploidies, an increased distance (>0.5) between red and blue lines on one parent's haplarithm suggests a lower chromosomal contribution from that parent, while a decreased distance (<0.5) on the other parent's haplarithm indicates a higher chromosomal contribution from that parent. A comprehensive visual guide for interpreting haplarithm plots is detailed in [1]. The following panel provides a zoomed-in view of the chromosome containing the variant loci of interest. Haplotype blocks are shown, with dark and light blue representing paternal haplotypes and dark and light red representing maternal haplotypes. Dark blue/red indicates the paternal/maternal haplotype carrying the variant allele. Variant loci are marked by vertical orange dashed lines, and the inherited parental haplotypes for these loci are those crossed by the lines, allowing for the inference of the embryo's carrier status. At the bottom, conclusions for PGT-M and PGT-A analyses are provided.

1. Zamani Esteki, M. *et al.* Concurrent Whole-Genome Haplotyping and Copy-Number Profiling of Single Cells. *Am J Hum Genet* **96**, 894 (2015).

A ONT1-E02

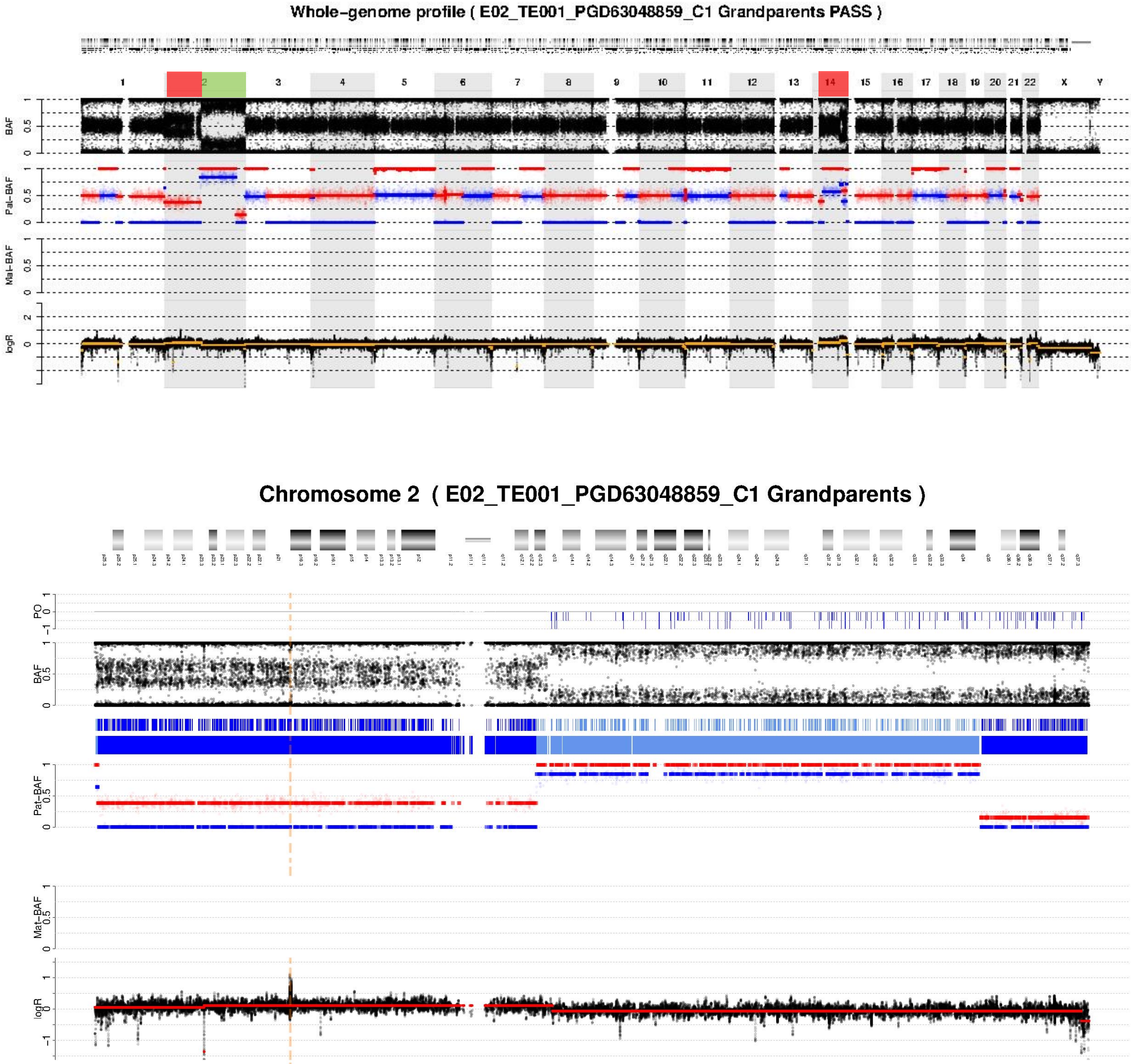

PGT-M:
Paternal SNV in *MSH2*: inherited

PGT-A:
Chromosome 2 mosaic duplication and deletion;
Chromosome 14 mosaic duplication

B ONT1-E03

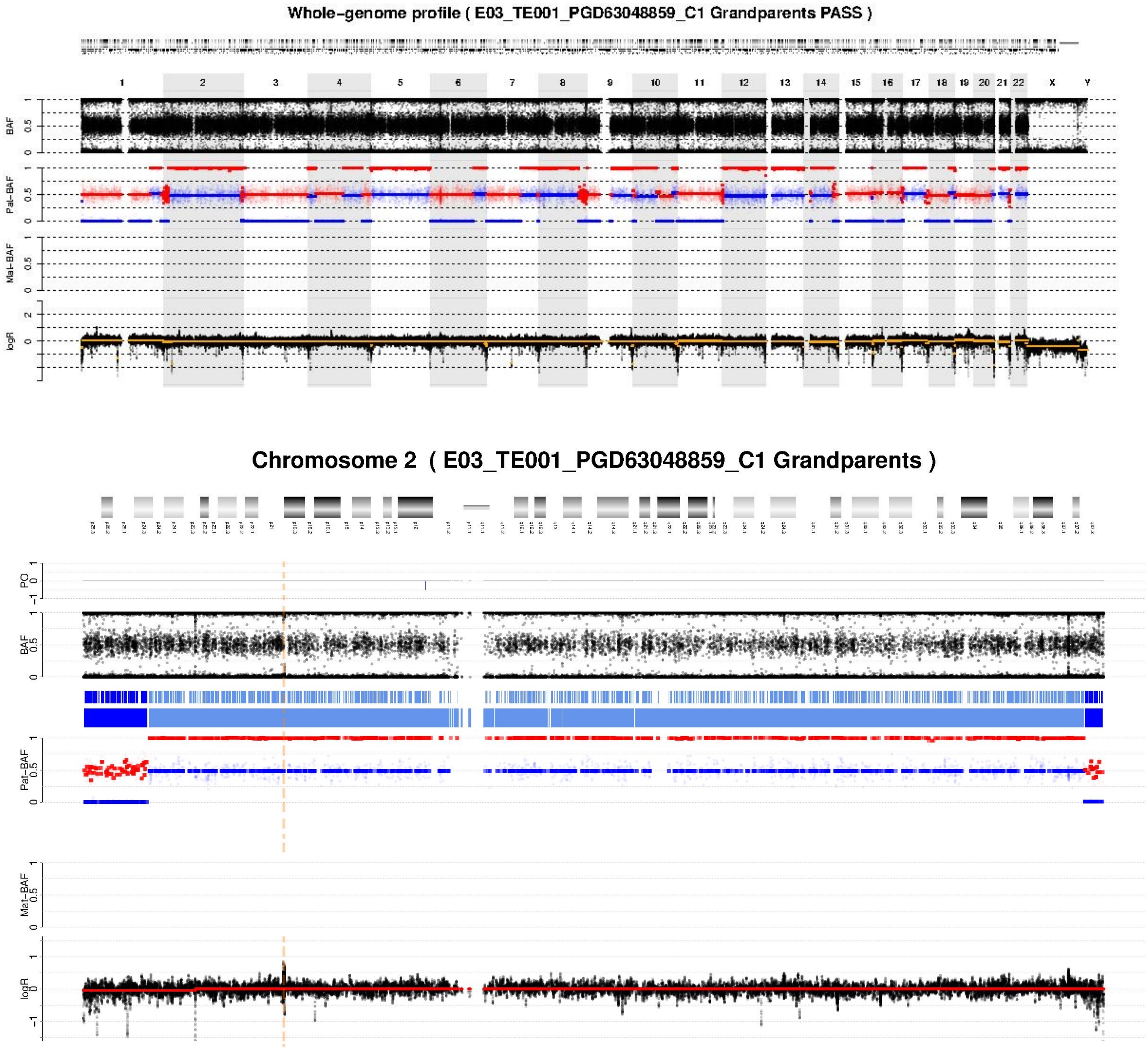

PGT-M:
Paternal SNV in *MSH2*: not inherited

PGT-A:
Euploid

C ONT2-E04

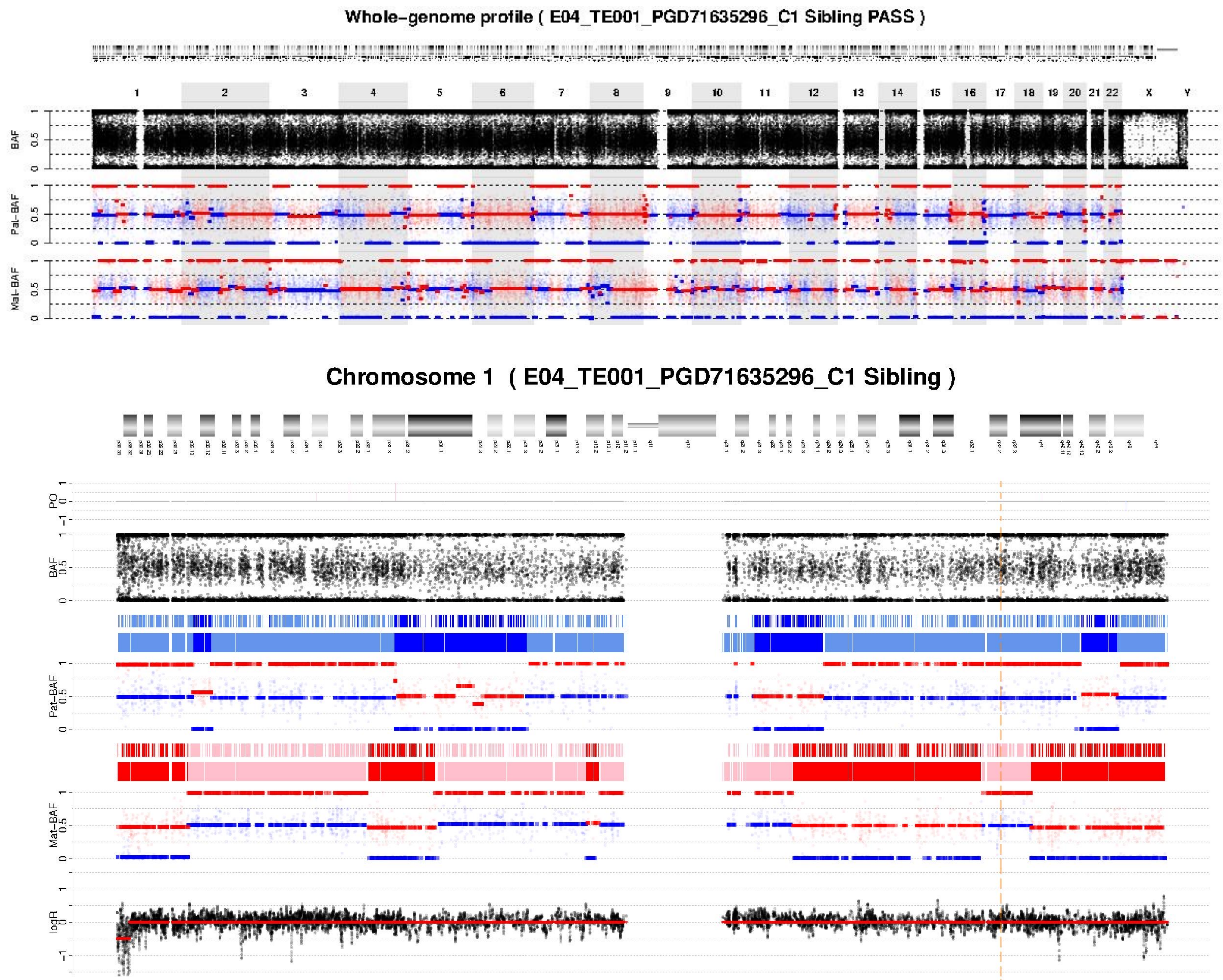

PGT-M:
Paternal SNV in *LAMB3*: not inherited
Maternal indel in *LAMB3*: not inherited

PGT-A:
Euploid

D ONT2-E06

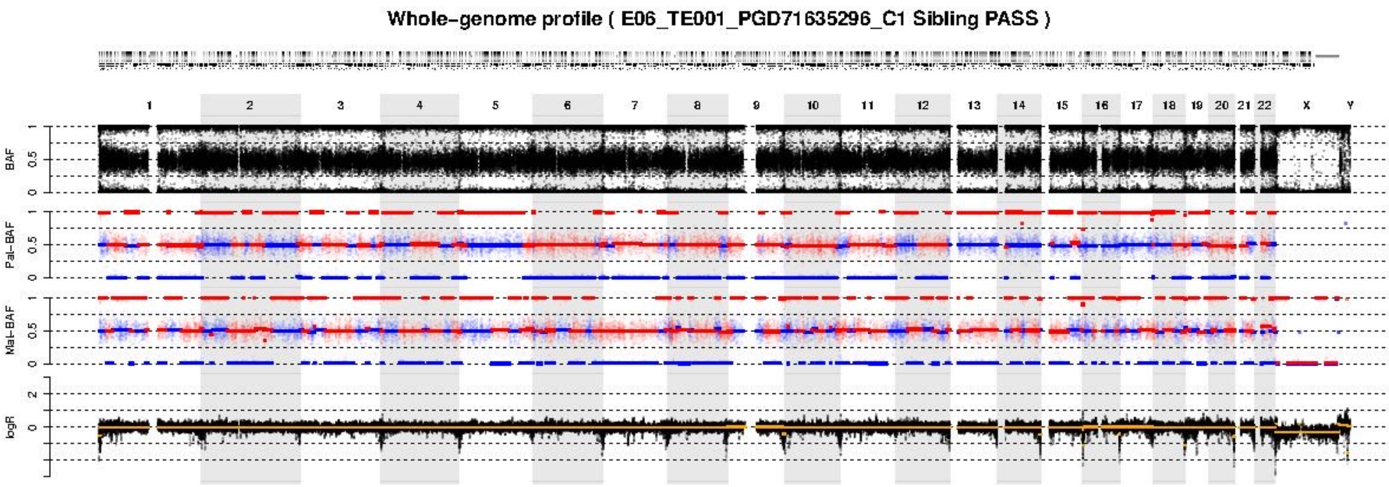

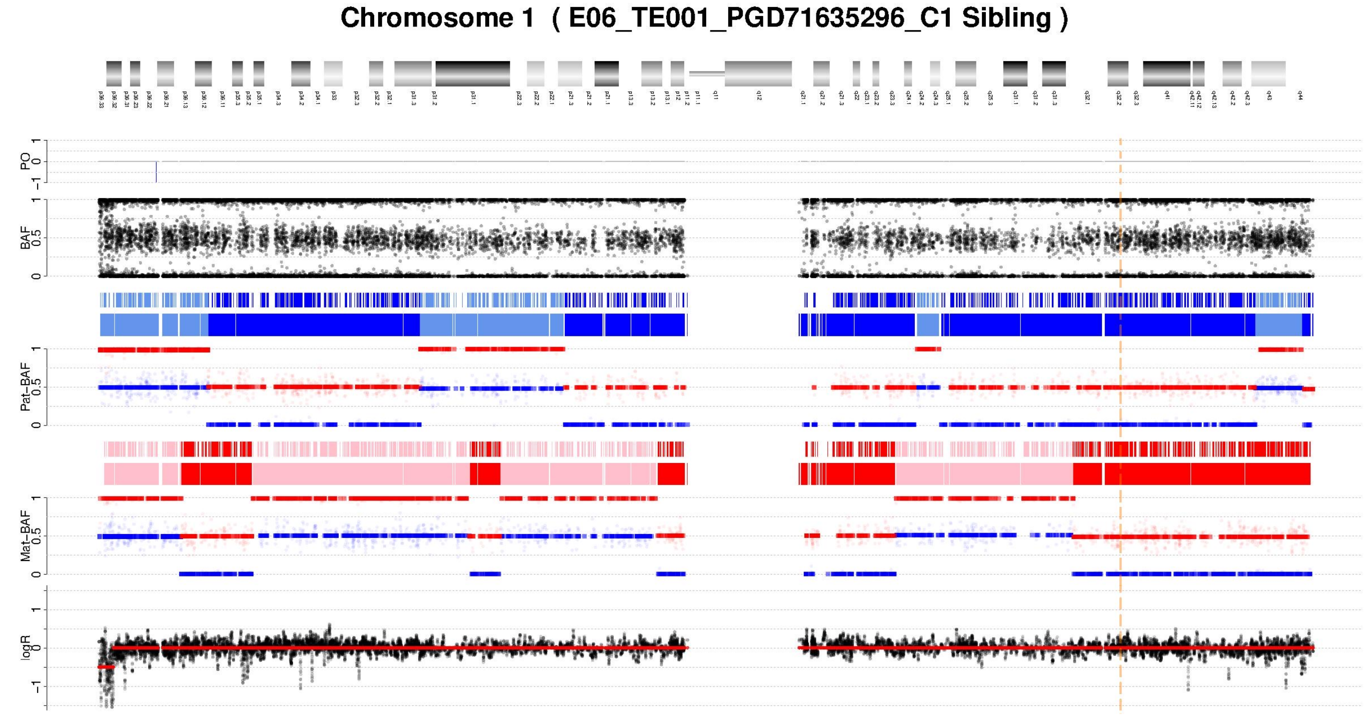

PGT-M:
Paternal SNV in *LAMB3*: inherited
Maternal indel in *LAMB3*: inherited

PGT-A:
Euploid

E ONT2-E20

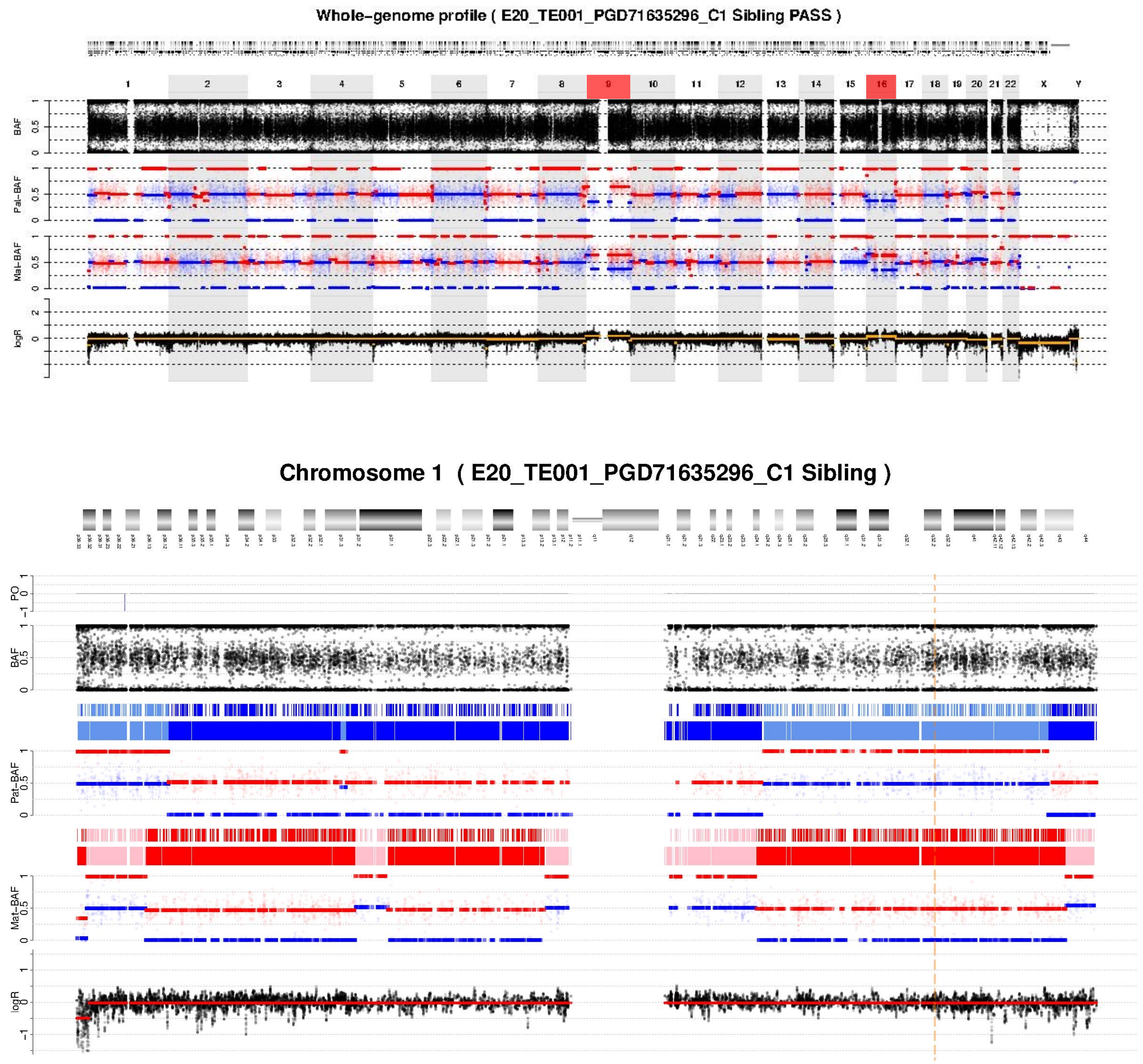

PGT-M:
Paternal SNV in *LAMB3*: not inherited
Maternal indel in *LAMB3*: inherited

PGT-A:
Trisomy for chromosomes 9 and 16, each involving an extra maternal chromosome resulting from nondisjunction during maternal meiosis I.

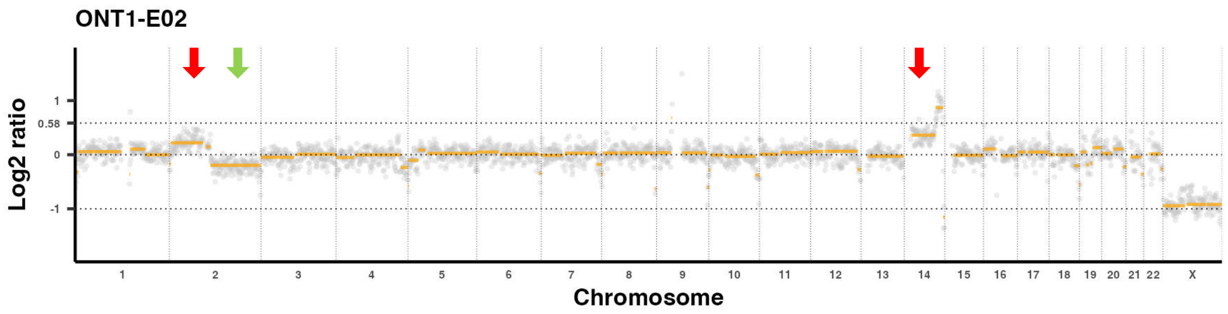

Supplementary Figure 10. PGT-A analysis using lrWGS data identified mosaic chromosomal abnormalities in ONT1-E02. The copy number plot reveals mosaic duplication of the short arm of chromosome 2 (indicated by a green arrow) and mosaic deletion of the long arm (indicated by a red arrow). Additionally, mosaic duplications are observed on chromosome 4 (indicated by a red arrow).

Supplementary Figure 11. Downsampling analysis of aneuploidy profiling for **(A)** ONT1-E02 and **(B)** ONT2-E20. For each embryo, the mapped BAM file was downsampled to coverage levels of 1x, 0.75x, 0.5x, 0.25x, 0.1x, 0.05x, and 0.01x, followed by aneuploidy profiling using the downsampled files.

A

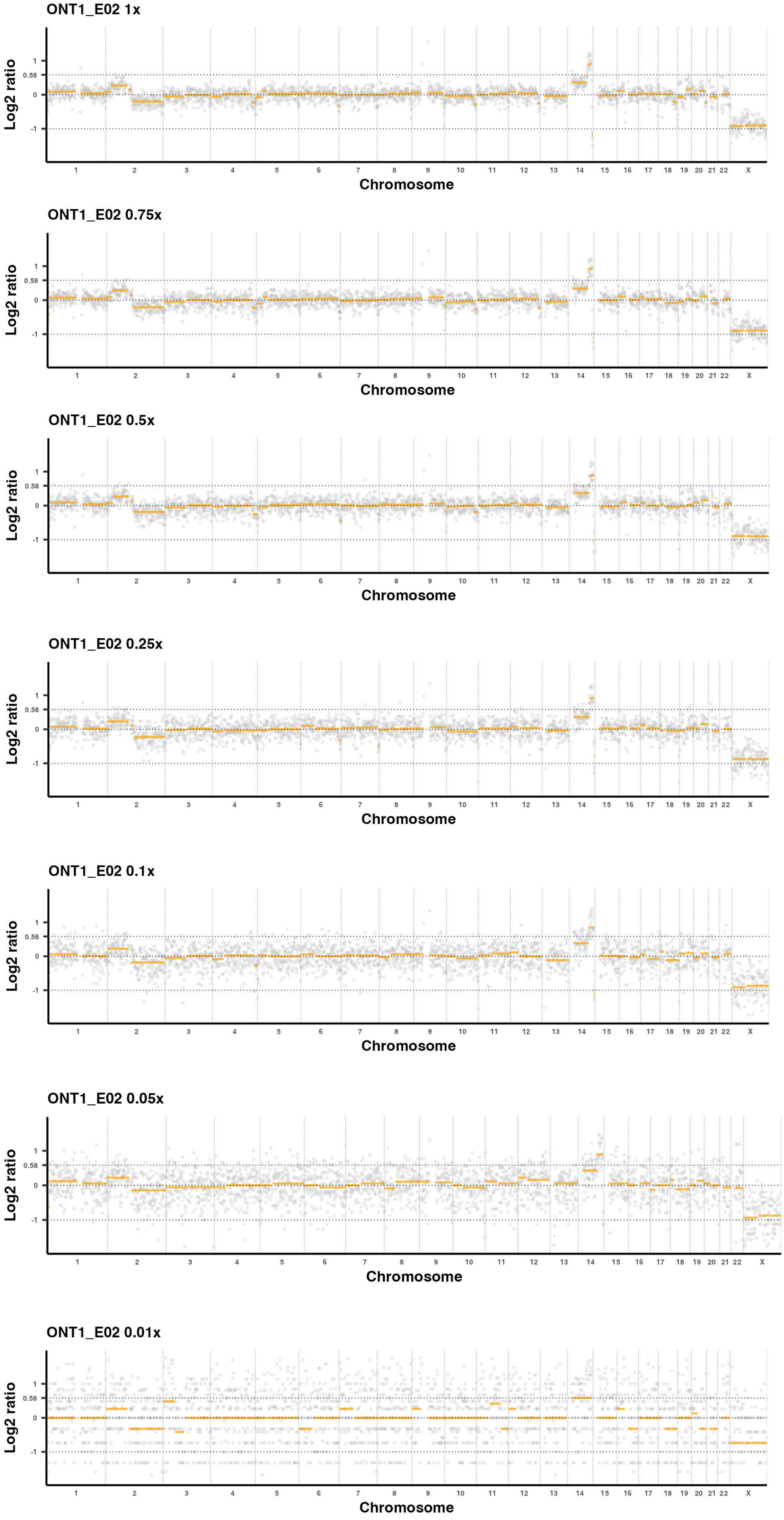
ONT1_E02 1x
ONT1_E02 0.75x
ONT1_E02 0.5x
ONT1_E02 0.25x
ONT1_E02 0.1x
ONT1_E02 0.05x
ONT1_E02 0.01x
Log2 ratio
1
0.58
0
-1
1 2 3 4 5 6 7 8 9 10 11 12 13 14 15 16 17 18 19 20 21 22 X
Chromosome

B

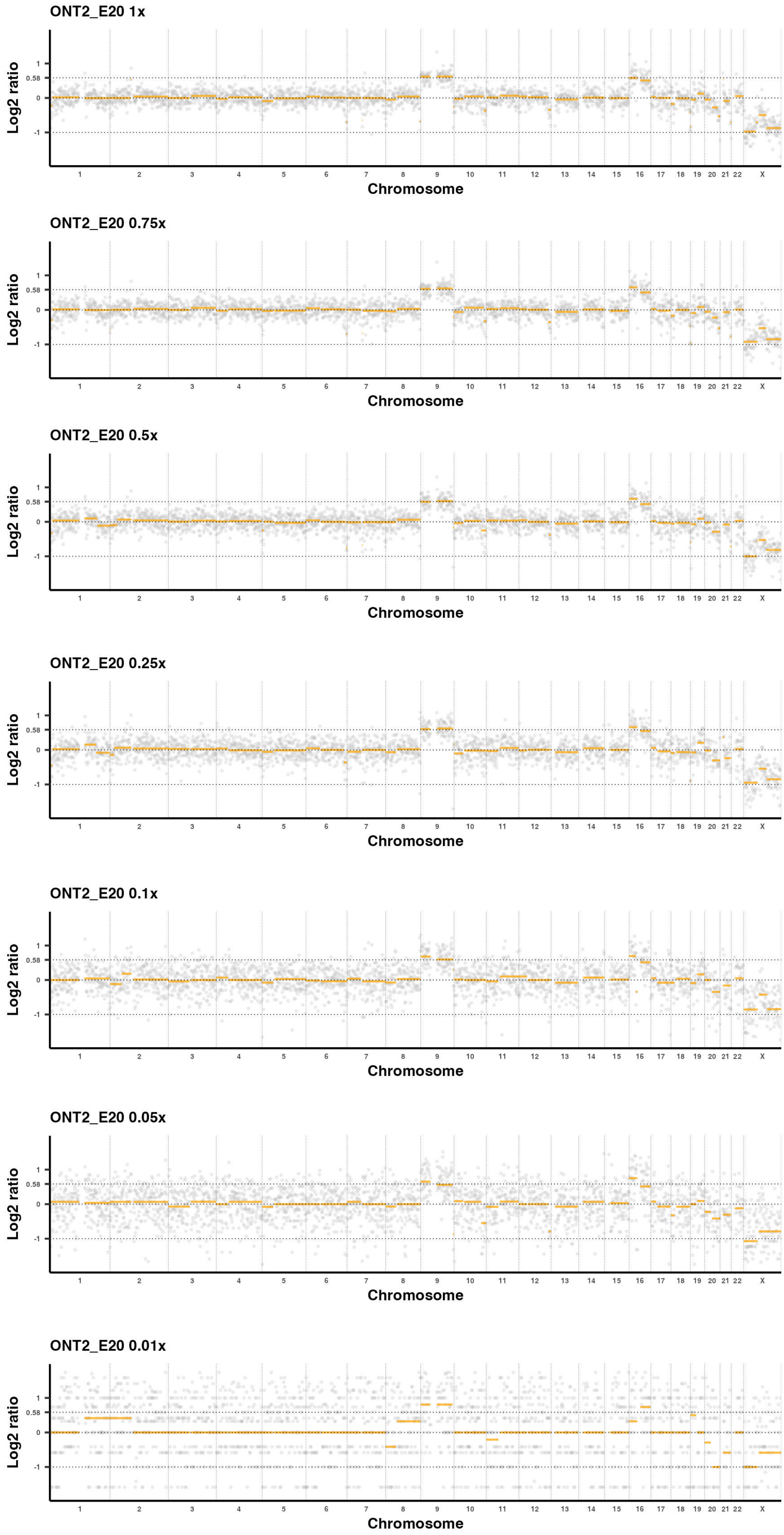
ONT2_E20 1x
ONT2_E20 0.75x
ONT2_E20 0.5x
ONT2_E20 0.25x
ONT2_E20 0.1x
ONT2_E20 0.05x
ONT2_E20 0.01x
Log2 ratio
1
0.58
0
-1
Chromosome
1 2 3 4 5 6 7 8 9 10 11 12 13 14 15 16 17 18 19 20 21 22 X
